# Clec12a inhibits MSU-induced immune activation through lipid raft expulsion

**DOI:** 10.26508/lsa.202301938

**Published:** 2023-06-20

**Authors:** Ying Xu, Dingka Song, Wei Wang, Shixin Li, Tongtao Yue, Tie Xia, Yan Shi

**Affiliations:** 1 Institute for Immunology, School of Medicine, Tsinghua University , Beijing, China; 2 State Key Laboratory of Oncogenes and Related Genes, Center for Single-Cell Omics, School of Public Health, Shanghai Jiao Tong University School of Medicine, Shanghai, China; 3 Institute of Coastal Environmental Pollution Control, Key Laboratory of Marine Environment and Ecology, Ministry of Education, Ocean University of China, Qingdao, China; 4 Joint International Research Laboratory of Agriculture and Agri-product Safety of the Ministry of Education, Yangzhou University, Yangzhou, China; 5 Department of Microbiology, Immunology and Infectious Diseases, Snyder Institute, University of Calgary, Calgary, Canada

## Abstract

Monosodium uric acid (MSU) crystal, the etiological agent of gout, has been shown to trigger innate immune responses via multiple pathways. It is known that MSU-induced lipid sorting on plasma membrane promotes the phosphorylation of Syk and eventually leads to the activation of phagocytes. However, whether this membrane lipid-centric mechanism is regulated by other processes is unclear. Previous studies showed that Clec12a, a member of the C-type lectin receptor family, is reported to recognize MSU and suppresses this crystalline structure-induced immune activation. How this scenario is integrated into the lipid sorting-mediated inflammatory responses by MSU, and particularly, how Clec12a intercepts lipid raft-originated signaling cascade remains to be elucidated. Here, we found that the ITIM motif of Clec12a is dispensable for its inhibition of MSU-mediated signaling; instead, the transmembrane domain of Clec12a disrupts MSU-induced lipid raft recruitment and thus attenuates downstream signals. Single amino acid mutagenesis study showed the critical role of phenylalanine in the transmembrane region for the interactions between C-type lectin receptors and lipid rafts, which is critical for the regulation of MSU-mediated lipid sorting and phagocyte activation. Overall, our study provides new insights for the molecular mechanisms of solid particle-induced immune activation and may lead to new strategies in inflammation control.

## Abstract (precis)

C type lection receptor Clec12a inhibits MSU induced Dendritic cell activation via its transmembrane domain rather than intracellular ITIM.

## Introduction

Lipid raft composed of tightly packed lipid molecules is critical for membrane-initiated signaling in eukaryotic cells (Sezgin et al, 2017). Previous studies highlighted the importance of lipid rafts in regulating various biological processes (Simons & Ikonen, 1997; Varshney et al, 2016). Relocation of membrane receptors in and out of the lipid raft region upon ligand recognition is an essential mechanism in controlling activation and subsequent signal transduction. Recently, the concept of liquid-ordered (Lo) and liquid-disordered (Ld) domains have been raised by membrane biologists. The composition and dynamic behavior of the Lo domain mimic lipid rafts and make it rather interesting to investigating the biological role of phase separation on plasma membrane in receptor-mediated signal transduction (van der Goot & Harder, 2001; Quinn & Wolf, 2009). Of immunological interest, the translocation of TCR into cholesterol-enriched lipid raft region is a prerequisite for T cell activation (Janes et al, 2000). In innate immunity, it is also reported that the activation of pattern recognition receptors requires the involvement of lipid rafts (Ruysschaert & Lonez, 2015). Reciprocally, some transmembrane proteins are capable of affecting the dynamics and aggregation of lipid molecules on the plasma membrane as well (Devaux, 1992; McIntosh et al, 2003; Corradi et al, 2018). The plasma membrane has a highly hydrophobic core, and the presence of sphingolipids, such as sphingomyelin, along with omnipresent cholesterol, creates an even and high degree of horizontal packing (Diaz-Rohrer et al, 2014). This design leads to preferential selection of transmembrane domains (TMD) of cell surface proteins. The composition, arrangement, and chemical property of amino acid residues in TMDs are also critical in determining repulsion and attraction towards various lipid species, hence affecting the behaviors of surrounding lipid molecules (Lorent & Levental, 2015). How such interactions influence intracellular signals is an emerging field of investigation.

Lipid rafts can participate in transmembrane signaling in multiple ways. For instance, GPI-anchored cell surface molecules, without any intrinsic signaling moieties, can lead to intracellular kinase activation by virtue of its association with the membrane lipids (lipid rafts) (Štefanová et al, 1991). For Group I metabotropic glutamate receptors, their association with lipid rafts drives caveolin (a component of a subset of lipid rafts)-dependent intracellular Ca^2+^ signaling (Roh et al, 2014). Therefore, lipid rafts can be seen as a conduit or a platform for organizing cell surface-signaling events. Our previous work showed that “lipid sorting” on plasma membrane upon monosodium urate crystal (MSU) binding recruits cholesterol-enriched microdomains on the MSU-binding site (Ng et al, 2008), promoting the engagement of immune tyrosine activating motif (ITAM)-containing molecules, either in the form of nondiscriminatory gathering of ITAM-containing transmembrane proteins or the intracellular protein moesin that has a cryptic ITAM at its N-terminus, to the plasma membrane (Mu et al, 2018). In addition, Alum, the commonly used adjuvant in vaccines, was also shown to be active in regulating lipid domain composition on innate immune cells to exert its adjuvant effects (Flach et al, 2011).

As solid structure recognition is an ancient mechanism predating immune receptors (Mu et al, 2018), our proposal provides a generic mechanism for phagocyte interaction with almost an infinite number of solid structures. Although no protein receptor is known to be responsible for MSU binding and immune activation, it was reported that the immune tyrosine-inhibiting motif (ITIM)-containing C-type lectin receptor Clec12a recognizes and inhibits MSU-induced inflammatory signals (Neumann et al, 2014). With the ITIM motif on the intracellular domain, it was reasonable to speculate an ITIM-mediated inhibitory effect of Clec12a would work through dephosphorylation that offsets the non-receptor kinase activities, such as those from Syk or common Fc γ chain (Lahoud et al, 2009). If this were true, it would represent one of the least studied signaling cascades, that a lipid sorting-based ITAM activation event was countered by a solid particle-specific receptor via its ITIM functions. However, whether this chain of events can take place has not been formally investigated.

Here, we report that Clec12a was indeed a negative regulator for MSU-mediated immune activation. However, the inhibitory activity of Clec12a was independent of its ITIM motif. In contrast with the prediction from previous studies (Lahoud et al, 2009), ITIM mutants exerted full suppressing capacity. Interestingly, we found that TMD of Clec12a was critically needed for the suppression. TMD domain swapping of Clec12a with several other Clec family members all showed a comparable level of suppressing efficacy, with the only exception of Clec4a. Imaging analysis indicated that whereas MSU crystals recruited lipid raft domains upon contact, the presence of Clec12a TMD drove their dispersion, whereas Clec4a TMD failed to show this effect. As an additional piece of evidence, Clec12a appeared to reduce silica-induced lipid raft association on live cells if this molecule was pulled into the lipid rafts via an artificial link on the silica surface, leading to depressed immune responses. Collectively, these data revealed a critical role of Clec12a TMD in disrupting MSU-induced lipid domain aggregation and downstream immune activation, providing new insights for future studies on immune recognition of solid particles.

## Results

### ITIM is not involved in Clec12a-mediated suppression of inflammatory response to MSU

**Figure S1.**
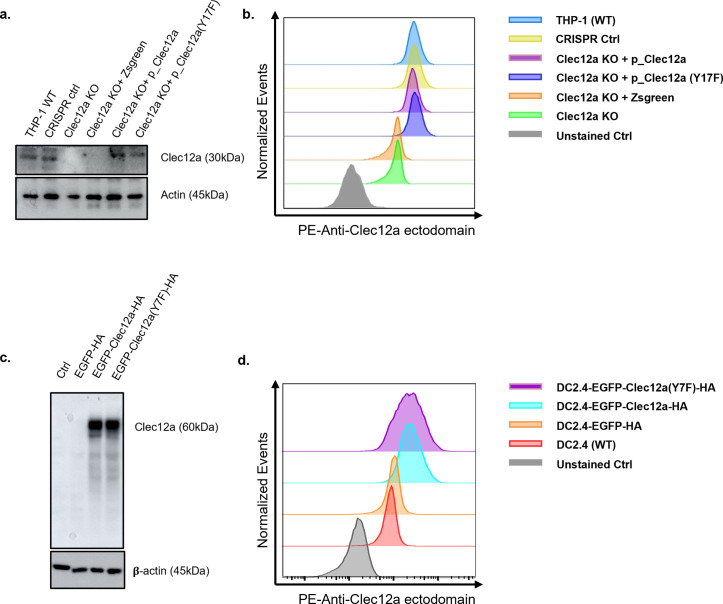
Generation and characterization of Clec12a mutations. **(A)** Western blot verification of Clec12a expression in various THP-1 cell lines. **(B)** Surface expression of Clec12a in various THP-1 cell lines were detected by staining with PE-conjugated anti-human Clec12a ectodomain antibody and flow cytometry analysis. **(C)** Western blot verification of Clec12a expression in DC2.4 cells. **(D)** As in (B), surface expression of Clec12a in various DC2.4 cell lines were detected by staining with PE-conjugated anti-mouse Clec12a ectodomain antibody and flow cytometry analysis. Data are represented from three independent experiments.

**Figure 1.**
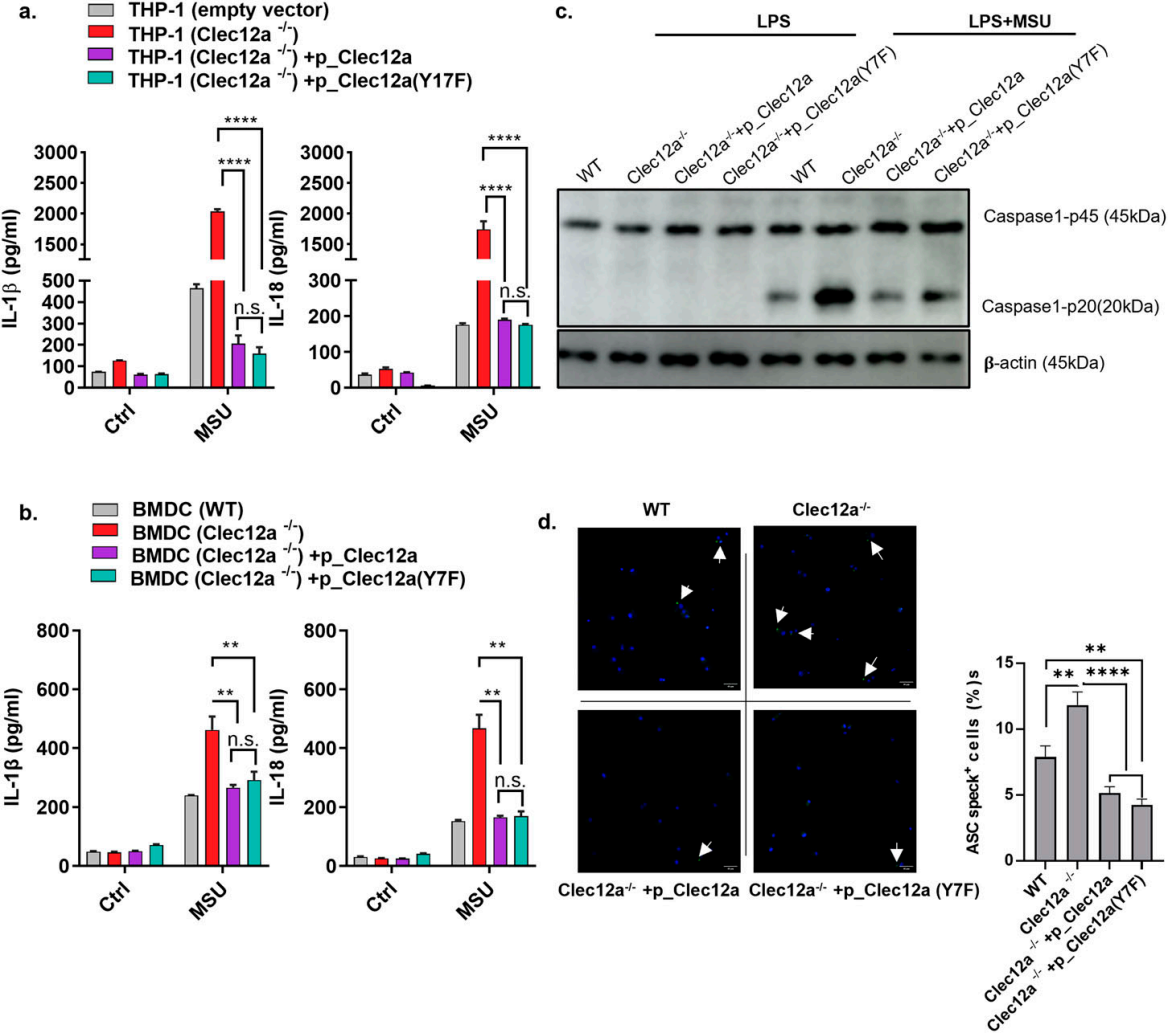
Clec12a attenuates monosodium uric acid (MSU)-induced inflammasome activation independent of immune tyrosine-inhibiting motif. **(A)** THP-1 cells were stimulated as indicated; 30 min later, the supernatants were discarded and replaced with fresh medium and cells were cultured for another 12 h. Later, IL-1β and IL-18 secretions were detected by ELISA. **(B)** Same as with BMDCs were used in place of THP-1. **(C)** BMDCs were stimulated with LPS and MSU crystals. 6 h later, Caspase 1 cleavage in cell lysates was analyzed by Western blot. **(D)** BMDCs were stimulated with LPS for 3 h, and then MSU for another 1 h. Cells were washed, fixed, permeabilized, and stained with FITC-conjugated anti-ASC Ab and DAPI. ASC speck formation was visualized under fluorescent microscope. Wavelengths of Ex were set at 405 nm (DAPI) and 561 nm (ASC), respectively; wavelengths of Em were set at 410–556 nm (DAPI) and 566–691 nm (ASC), respectively. Magnification of objective lens: 10x. Left panel: pictures were processed and pseudo-color images were generated by Image J (blue: DAPI; green: ASC). White arrows indicate ASC specks. Scale bar: 40 μm. Right panel: statistical analysis of ASC speck positive cells in each group. Data are represented from of three independent experiments. WT, wild type; BMDC, bone marrow dendritic cells; IL, interleukin; LPS, liposaccharide. ASC, apoptosis-associated speck-like protein containing a caspase-recruitment domain (CARD); FITC, fluorescein isothiocyanate; MSU, monosodium uric acid; DAPI, 4′,6-diamidino-2-phenylindole; Ex, excitation; Em, emission.

**Figure S2.**
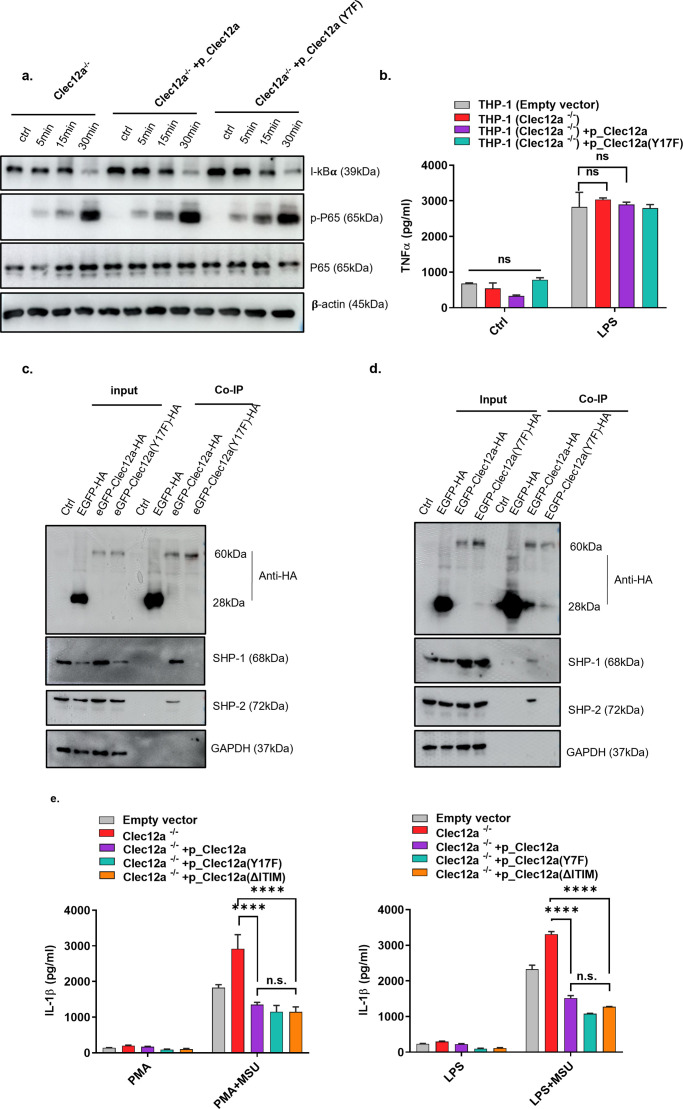
Clec12a attenuates monosodium uric acid-induced inflammasome activation independent of immune tyrosine-inhibiting motif (ITIM) motif. **(A)** Clec12a^−/−^ mouse BMDCs restored with WT or ITIM mutation (Y7F) version of exogenous Clec12a were stimulated with LPS, NF-κB activation was detected at different time points as indicated. **(B)** Control THP-1 cells, Clec12a deficient THP-1 cells, Clec12a-deficient THP-1 cells overexpressed with WT or ITIM mutant (Y17F) Clec12a were stimulated with LPS, TNF-α secretion was detected 12 h after LPS stimulation. **(C)** Co-immunoprecipitation detection of SHP1/2 with Clec12a in THP-1 cells. **(D)** Co-immunoprecipitation detection of SHP1/2 with Clec12a in DC2.4 cells. **(E)** Similar to Fig 1A and B, except the full ITIM truncation versions of Clec12a were added for comparison. 12 h later, IL-1β secretion in THP-1 (left panel) and BMDC (right panel) were accessed. Data are represented from of three independent experiments.

As myeloid cells secrete interleukin 1beta (IL-1β) in response to MSU stimulation (Chen et al, 2006), the effects of Clec12a-mediated suppression can be assessed by the diminished IL-1β production. To establish a clean background, we used CRISPR/Cas9 to produce diploid deletion of *clec12a* gene in human monocyte THP-1 cells. The efficacy of Clec12a depletion was verified by Western blot (Fig S1A). The expression of Clec12a was then reestablished by lentiviral transfection (Fig S1A). As Clec12a has an intracellular ITIM domain responsible for SH2 domain-containing phosphatase 1/2 (SHP-1/2) recruitment and potentially transducing inhibitory effect, the ITIM mutant version of Clec12a (Y17F) was also generated (Fig S1A). Surface expression of Clec12a constructs were validated by anti-Clec12a Ab staining and flow analysis (Fig S1B). As expected, depletion of endogenous Clec12a-enhanced IL-1β and IL-18 production in response to stimulation of MSU, and overexpression of WT Clec12a significantly reduced the secretion of those cytokines. Unexpectedly, overexpression of the Clec12a ITIM mutant (Y17F) exhibited a comparable suppressive effect to WT (Fig 1A). To rule out that it was a peculiarity in THP-1 cells, we used similar overexpressing approaches on BMDCs from *clec12a*^−/−^ mice. With those primary cells, ITIM mutation (Y7F) did not affect the inhibitory activity of Clec12a in response to MSU as well (Fig 1B). Accordingly, deletion of Clec12a led to increased production of mature Caspase-1 (p20), and the overexpression of either WT or Y7F mutant reinstated the suppressive effect (Fig 1C). This impact on inflammasome-associated p20 conversion likely reflected that lipid sorting-based signaling mechanism controls the intensity of phagocytosis, which is a prerequisite for intracellular NLRP3 inflammasome assembly. In line with this, the inhibitory efficacy on the intracellular apoptosis-associated speck-like protein containing a caspase recruitment domain (ASC) condensation (specks formation) after MSU stimulation was comparable between WT and Y7F mutant groups as well (Fig 1D). However, the absence or presence of any versions of Clec12a did not affect NF-κB activation (Fig S2A), which in the case of BMDCs, indicated the priming phase (signal 1) for inflammasome activation. Similarly, little impact on TNFα production in response to bacterial endotoxin LPS was observed in all versions of Clec12a transfectants (Fig S2B), suggesting that Clec12a is not involved in signal I activation, which is the prerequisite for inflammasome activation.

All these data implicated the possibility that the suppressive activity of Clec12a on MSU-induced inflammasome activation was independent of ITIM. To ascertain this point, HA-tagged WT or ITIM mutant Clec12a (Y7F) were overexpressed in murine DC line DC2.4 cells. The baseline expression of Clec12a on DC2.4 cells and the expression levels of various Clec12a constructs were assessed (Fig S1C). Notably, the baseline expression of Clec12a on DC2.4 cells is nearly undetectable and the expression levels of WT and ITIM mutant Clec12a were equivalent (Fig S1C). The surface expression of various Clec12a constructs on DC2.4 cells was further validated by flow cytometry (Fig S1D). Using EGFP-HA as the efficacy control, the interaction of Clec12a with SHP-1/2 was studied by co-immunoprecipitation (Co-IP). It showed that WT Clec12a was able to Co-IP both SHP-1 and SHP-2, whereas neither was pulled down with ITIM mutant Clec12a (Fig S2C and D). To further validate the ITIM-independent role of Clec12a inhibiting MSU-induced inflammasome activation, the full ITIM truncation version of Clec12a (Clec12a [ΔITIM]) was introduced and IL-1β secretion was assessed upon MSU stimulation. The overexpression of Clec12a (ΔITIM) significantly inhibits MSU-induced L-1β secretion in both mouse BMDC and THP-1 cells, which was comparable with the single amino acid mutation version of Clec12a ITIM domain (Fig S2E). These data indicate that the intrinsic association between ITIM and SHP-1, which mediates the bulk of suppression activities in other scenarios involving CLRs, was operational in our system. However, this dephosphorylation event was not used by Clec12a to reduce MSU-mediated inflammatory effects.

### ITIM-independent role of Clec12a in attenuating MSU induced DC activation

**Figure 2.**
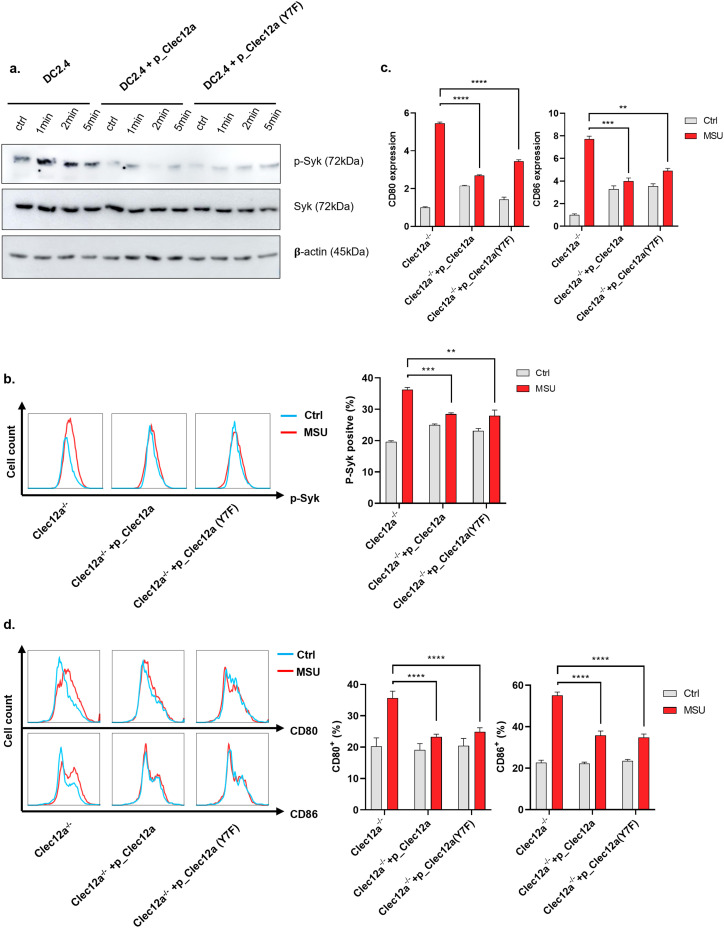
Immune tyrosine-inhibiting motif-independent role of Clec12a in inhibiting monosodium uric acid (MSU)-induced DC activation. **(A)** DC2.4 cells were stimulated with MSU for the indicated time, Syk phosphorylation was assessed by Western blot. **(B)** DC2.4 cells were stimulated with MSU for 10 min; cells were then washed, fixed, permeabilized, and stained with anti-p-Syk antibody for flow analysis. Left panel: peak shift of p-Syk fluorescent signal revealed by flow cytometry; right panel: statistical analysis of the percentage of p-Syk-positive cells (fluorescent intensity cut-off value: 1 × 10^3^). **(C)** BMDCs were stimulated with MSU, 6 h later, CD80 and CD86 mRNA analyzed RT–PCR. The value of Ctrl *clec12a*^−/−^ cells was set as one and all other groups were normalized accordingly. **(D)** As in (C), CD80/86-positive cells were analyzed by flow cytometry. Data are represented from of three independent experiments. Ctrl, Control.

MSU is an endogenous danger signal in inducing inflammatory response (Shi et al, 2003). MSU-mediated phagocyte activation is not limited to NLRP3 inflammasome, it was shown that lipid raft aggregation in response to solid particle binding including MSU led to tyrosine protein kinase Syk phosphorylation (Ng et al, 2008), which, along with Zap70, is the obligatory conduit for ITAM-based immune activation. We assessed whether Clec12a negatively impacted Syk activation, which helped reveal its point of interception in MSU signaling cascade. The results showed that expression of either WT or Y7F mutant Clec12a in DC2.4 cells markedly reduced the level of p-Syk under MSU stimulation, suggesting that the blockage occurs before the Syk recruitment (Fig 2A and B). Expression of CD80/86, another set of surface markers reflecting DC activation, was also accessed. RT–PCR analysis revealed that the amounts of CD80/86 transcripts in *clec12a*^−/−^ BMDCs were substantially elevated upon MSU stimulation, yet overexpression of either WT or Y17F mutant Clec12a significantly reduced CD80/86 transcription (Fig 2C). Similar results were observed at the protein levels by surface CD80/86 staining and flow cytometry analysis (Fig 2D). Taken together, these results demonstrated that Clec12a can inhibit DC activation in response to MSU crystals in general.

### Clec12a interrupts MSU-induced lipid raft aggregation

**Figure 3.**
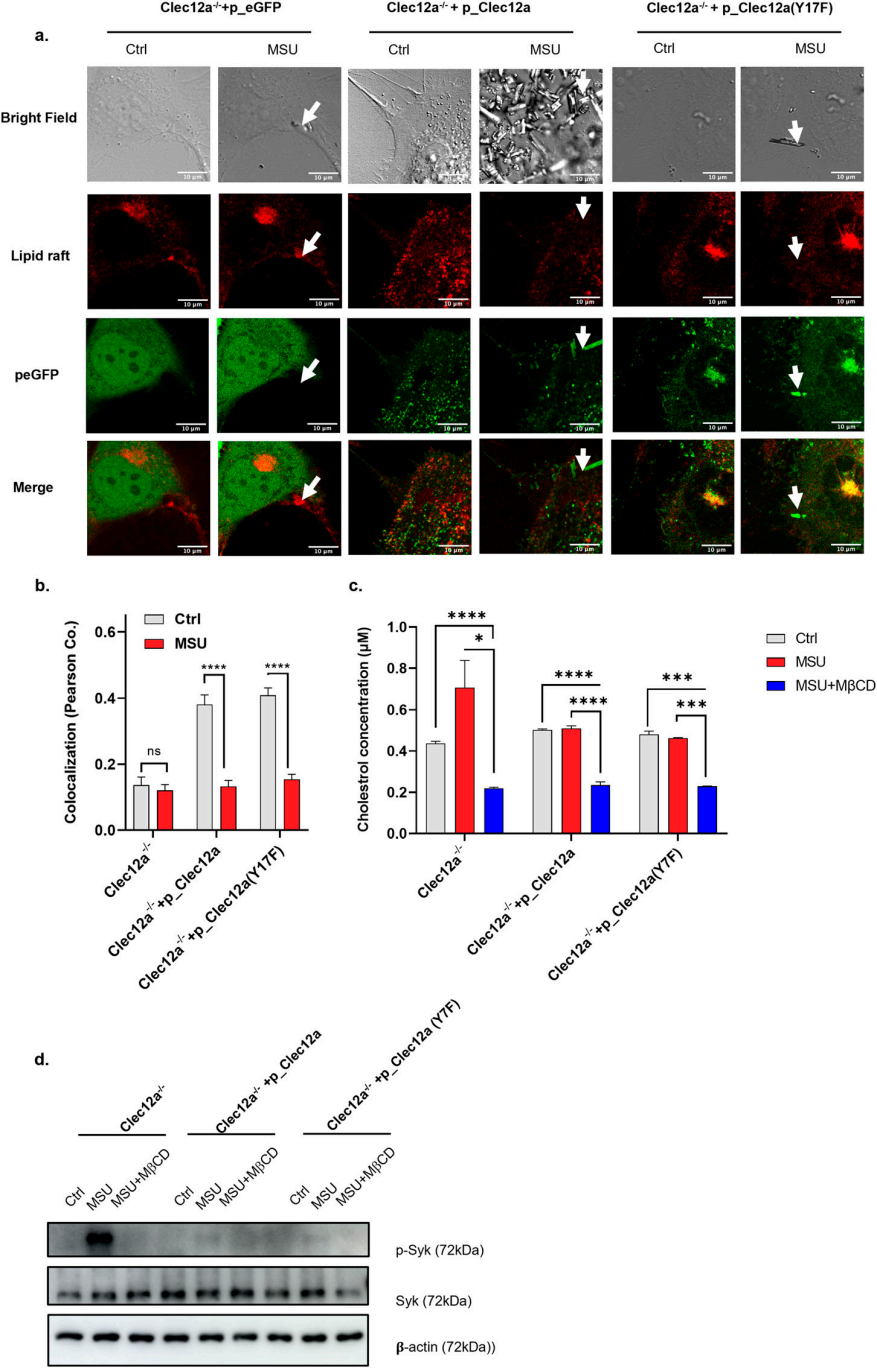
Clea12a disrupts monosodium uric acid (MSU)-induced “lipid sorting.” **(A)** Confocal images of the distribution of lipid raft (red) and Clec12a-eGFP (or empty vector) (green) on the plasma membrane. Endogenous Clec12a-deficient Hela cells overexpressing with the indicated vectors were stained with Alexa-Fluor 647-conjugated CTB, stimulated with MSU (or left untreated), and monitored under a confocal microscope. Wavelengths of Ex were set at 488 nm (eGFP) and 633 nm (AF647), respectively; wavelengths of Em were set at 493–561 nm (eGFP) and 688–756 nm (AF647), respectively. Bright field images were also captured for MSU visualization. Magnification of oiled-immersed objective lens: 63x. Images were processed by Image J, the BF images (first row), lipid raft (red, second row), eGFP (green, third), and merged images (fourth row) were displayed. Arrows indicate MSU-contacted regions on the plasma membrane. Scale bar: 10 μm. **(A, B)** Statistical analysis of the colocalization rate between lipid raft and Clec12a-eGFP in (A). **(C)** Efficiency of cholesterol depletion by MβCD. DC2.4 cells overexpressing with the indicated vectors were stimulated with MSU, MSU + MβCD or left untreated. Cells were washed with PBS for three times and the amount of retained cholesterol was detected. **(D)** DC2.4 cells overexpressing with the indicated vectors were stimulated with MSU, MSU + MβCD or left untreated, Syk phosphorylation was detected by Western blot. Data are represented from of three independent experiments. CTB, chlora toxin subunit B; MβCD, methyl-beta-cyclodextrin; Ctrl, control; BF, bright field.

**Figure S3.**
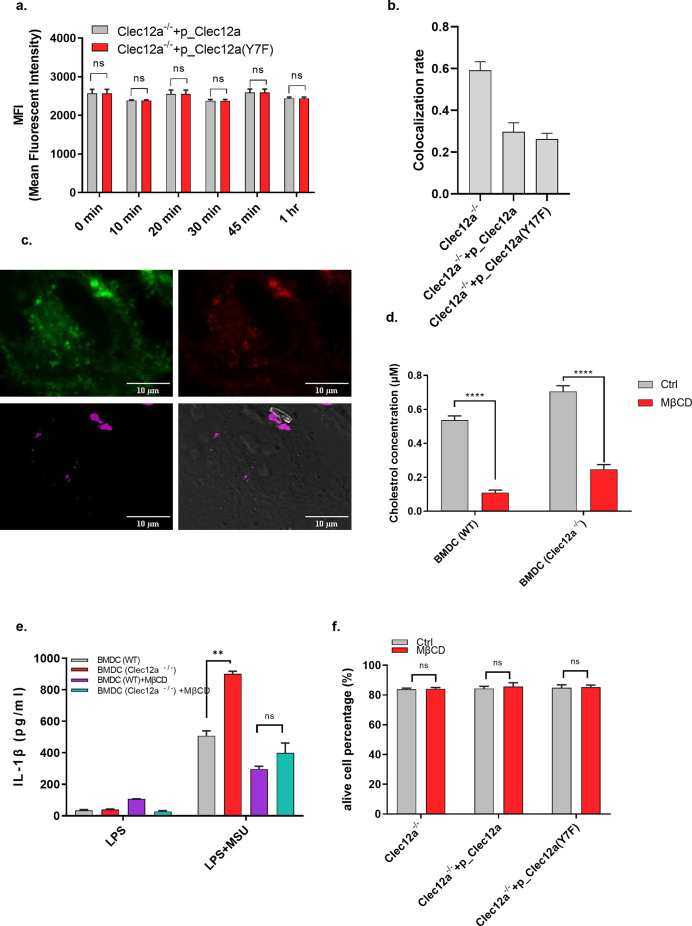
Clea12a disrupts monosodium uric acid (MSU)-induced “lipid sorting.” **(A)** Detection of Clec12a internalization after MSU stimulation. DC2.4 cells of different groups were stimulated with MSU crystals for various time lengths, then the cells were washed and stained with PE-conjugated anti-Clec12a domain antibody for flow cytometry analysis. The fluorescent intensity of PE signal indicates the amount of Clec12a retained on the plasma membrane. **(B)** Colocalization analysis of MSU and lipid raft. According to Fig 3A, the colocalization rate between MSU (Bright field) and lipid raft (red) of each group was calculated. **(C)** GP imaging upon MSU stimulation. Similar to Fig 3A, endogenous Clec12a-deficient Hela cells were stained with 5 μM Di-4-ANEPPDHQ in a serum-free medium for 30 min and washed three times with PBS. Samples were stimulated with MSU and monitored under a confocal microscope. The wavelength of Ex was set at 488 nm and the wavelengths of emission were set at 550–570 nm (Lo) and 640–660 nm (Ld), respectively. Bright field (BF) was also captured for MSU visualization. Magnification of oiled-immersed objective lens: 63x, Upper left: snapshot of Lo; upper right: snapshot of Ld, lower left: pseudo color image generated basing on GP values (purple color indicates high GP value); lower right: merged image of GP and BF. Scale bar: 10 μm. **(D)** Efficiency of cholesterol depletion by MβCD. Similar to Fig 3C, WT or Clec12a^−/−^ BMDC cells were treated with MβCD or left untreated. Cells were washed with PBS for three times and the amount of retained cholesterol was detected. **(E)** WT or Clec12a^−/−^ BMDC were stimulated with LPS + MSU in the presence or absence of MβCD. 12 h later, IL-1β secretion was detected by ELISA. **(F)** DC2.4 cells treated with MβCD for 1 h or left untreated were washed with PBS, replenished with cultural medium for an additional incubation of 5 h. Later, cell death was detected by Annexin-5/PI staining and flow cytometry analysis. Data are represented from of three independent experiments. Lo, liquid ordered; Ld, liquid disordered.

Lipid raft clustering either condenses ITAM-containing membrane receptors or recruits intracellular moesin to the inner leaflet of the plasma membrane, both of which provide ITAMs required for Syk membrane recruitment (Mu et al, 2018). To investigate the involvement of Clec12a in “MSU-lipid raft-Syk” signaling axis, endogenous Clec12a-deficient Hela cells were transfected with either GFP-fused WT Clec12a, Y17F mutant Clec12a, or an empty vector, and cells were stimulated with MSU and stained with cholera toxin B subunit (CTB) that specifically recognizes ganglioside GM1 for lipid raft visualization and colocalization analysis. Hela cells were used because they are non-phagocytic, permitting a steady imaging status without any phagocytic progression, while at the same time retaining the autonomous membrane events. In the presence of MSU stimulation, strong colocalization between MSU and lipid rafts was observed in the vector transfectant, which was consistent with previous reports (Fig 3A) (Ng et al, 2008). However, overexpression of either the WT or Y17F mutant Clec12a largely disrupted the association of MSU and lipid rafts, with a reciprocal-increased association between MSU and Clec12a (Fig 3A). Pearson’s coefficient in both WT and Y17F mutant Clec12a transfectant groups was significantly reduced compared with their unstimulated controls (Fig 3B). It has been reported that Clec12a is internalized upon MSU recognition (Gagné et al, 2013). To exclude the possibility of Clec12a internalization that might affect our results, we performed a time-course assay of MSU-induced Clec12a internalization by flow cytometry. It revealed that MSU stimulation does not induce significant endocytosis of Clec12a in 1 h (Fig S3A), which in addition, lagged behind the time of MSU stimulation in our experiment settings (5–10 min) and would not affect the interpretation to our results.

Ganglioside GM1 staining of CTB reflects a proportion rather than the total amount of lipid rafts on the plasma membrane, we specifically calculated the colocalization rate between MSU and CTB staining lipid rafts on different cell types. It shows that the colocalization rate between MSU and CTB-positive areas in *clec12a*^−/−^ cells is less than 1, suggesting the existence of CTB-negative lipid rafts. In addition, in the presence of WT or Y17F mutant Clec12a, the colocalization rate between MSU and CTB substantially reduced (Fig S3B). These results support our hypothesis that Clec12a suppresses MSU-induced lipid raft recruitment independent of its intracellular ITIM. In addition to CTB staining, other strategies were also developed for lipid raft probing (Carquin et al, 2014). To further verify lipid raft visualization by CTB staining in our study, we used another lipidic membrane fluorescent probe, di-4-ANEPPDHQ, for direct membrane staining and GP imaging analysis (Dinic et al, 2011). It revealed that the MSU-contacted region is associated with the Lo domain, indicating the recruitment of lipid rafts in the MSU-contacted place (Fig S3C).

To determine the role of Clec12a and lipid raft on MSU-induced downstream signals, Syk phosphorylation was studied on different cells upon MSU stimulation with or without MβCD treatment. It showed that MβCD efficiently depleted cholesterol on cells (Fig 3C). Furthermore, the enhanced Syk phosphorylation in the absence of Clec12a interference was abrogated by MβCD treatment, suggesting the Clec12a regulation was at the step of lipid organization (Fig 3D). On the other hand, the Syk phosphorylation was low in the presence of Clec12a, with or without MβCD. These data suggest that that interaction between Clec12a and MSU inhibited the lipid raft aggregation in response to MSU crystals (Fig 3D). In supporting this, MβCD treatment significantly suppressed IL-1β secretion in *clec12a*^−/−^ BMDCs in response to LPS plus MSU stimulation, demonstrating the dominant role of lipid rafts in transducing MSU-initiated pro-inflammatory signals (Fig S3D and E).

It has been also reported that MβCD treatment can affect the overall organization of the plasma membrane (Zidovetzki & Levitan, 2007), or even lead to the formation of solid ordered or gel domains (Giocondi et al, 2004). To exclude the cytotoxic effect of MβCD on cells, cell death events were analyzed after MβCD treatment. It shows that MβCD stimulation in our setting does not induce significant cell death (Fig S3F). Nevertheless, as MβCD treatment may cause reorganization of the plasma membrane beyond lipid raft disruption, our results of MβCD treatment can be used only as supporting but not definitive evidence for the involvement of lipid rafts. Collectively, these results highlight the role of lipid rafts in mediating Clec12a’s inhibiting role in MSU-induced DC activation.

### Clec12a TMD interferes with lipid raft sorting

**Figure 4.**
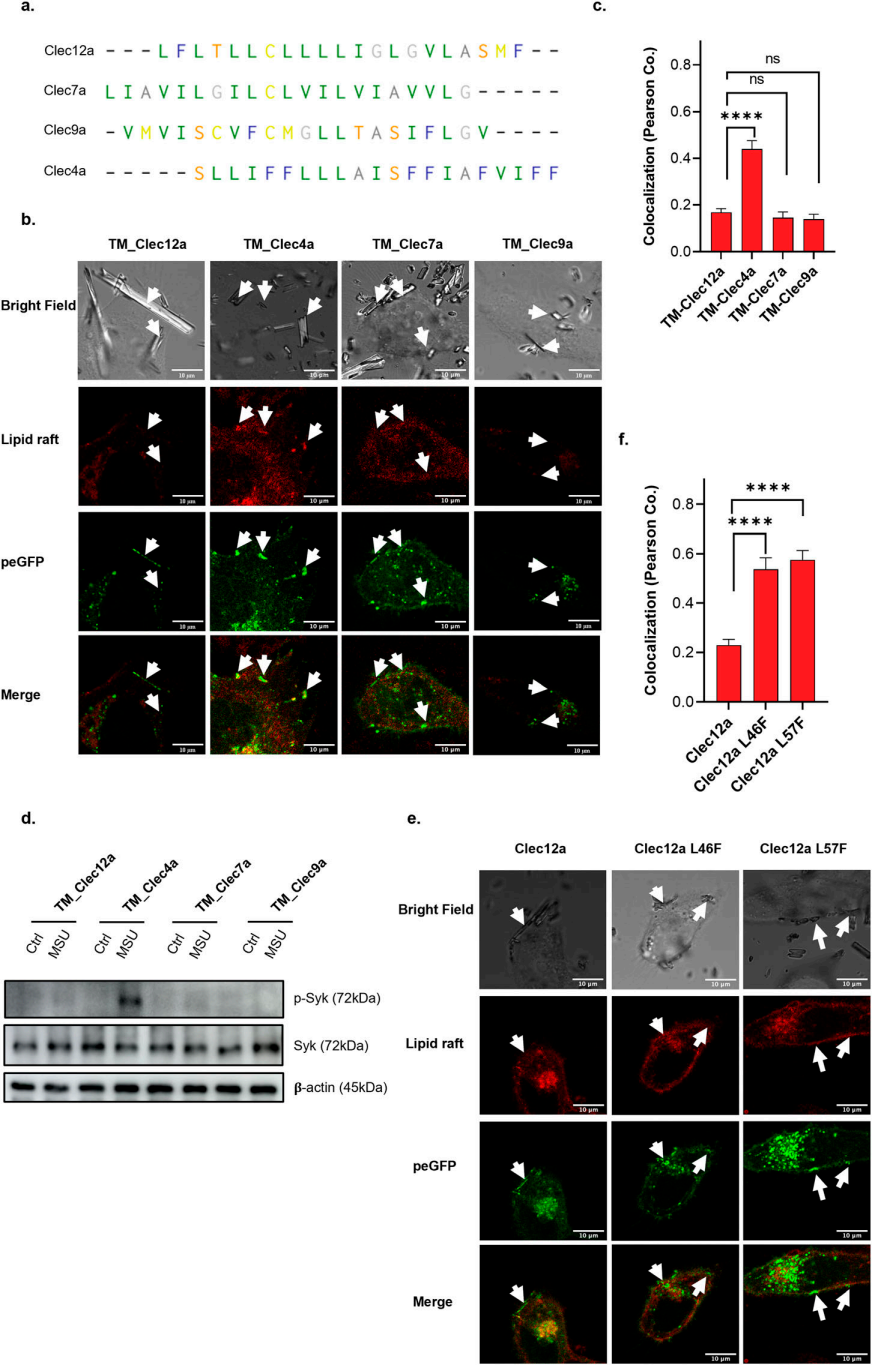
The transmembrane region of Clea12a is critical in the inhibition of monosodium uric acid (MSU)-induced lipid sorting. **(A)** Transmembrane sequence comparison of Clec12a and other family members. **(B)** As in Fig 3A, endogenous Clec12a-deficient Hela cells were overexpressed with different Clec12a transmembrane mutants and monitored under a confocal microscope. All parameters were set as same as Fig 3A. Scale bar: 10 μm. **(B, C)** Statistical analysis of the colocalization rate in (B). **(D)** As in Fig 3D, cells were transfected with different Clec12a mutants: the transmembrane domain (TM) of Clec12a swapped with the same region from Clec4a (TM_Clec4a) or Clec7a (TM_Clec7a) or Clec9a (TM_Clec12a) or kept unchanged (TM_Clec12a). Later, DC2.4 cells overexpressing different Clec12a transmembrane mutants were stimulated with MSU or left untreated. Syk phosphorylation was analyzed by Western blot. **(B, E)** Confocal imaging of Hela cells overexpressing WT or L46F, L57F mutation of Clec12a and stimulated with MSU as in (B). Scale bar: 10 μm. **(E, F)** Statistical analysis of the colocalization rate in (E). Data are represented from of three independent experiments. TM, transmembrane; L, leucine; F, phenylalanine.

**Figure S4.**
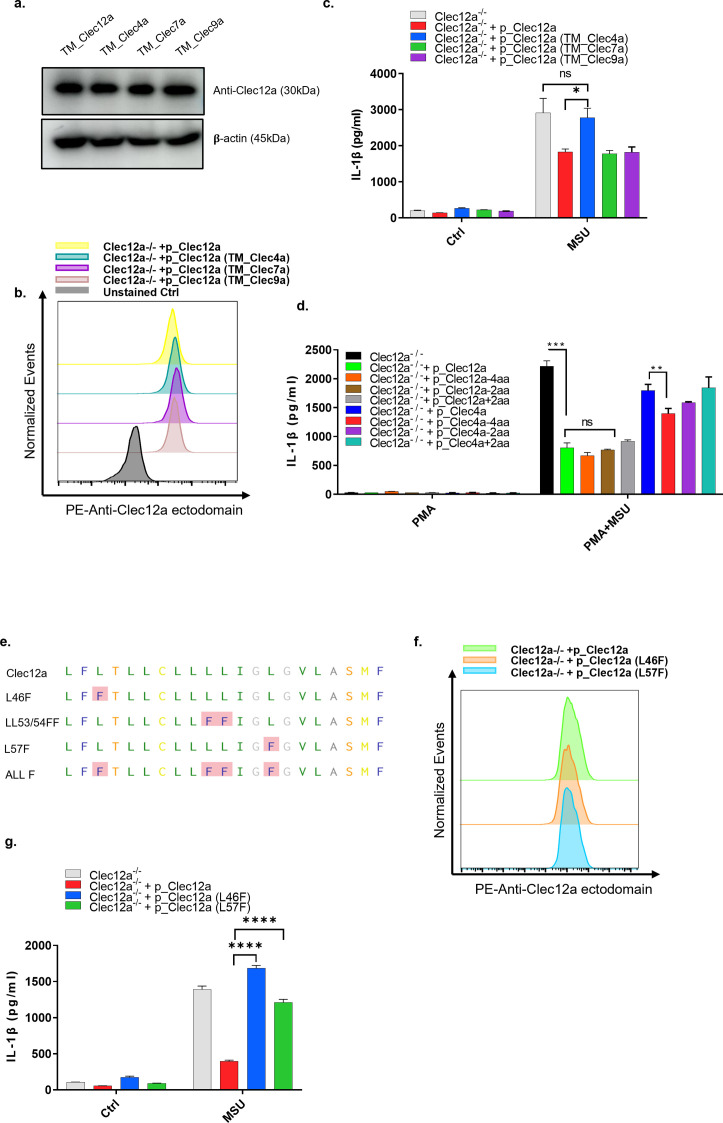
The transmembrane region of Clea12a is critical in the inhibition of monosodium uric acid-induced lipid sorting. **(A)** Western blot verification of the expression of different transmembrane domain mutants of Clec12a on THP-1 cells. **(B)** THP-1 cells with various transfectants of Clec12a TMD mutants were stained with PE-conjugated anti-Clec12a ectodomain antibody and detected by flow cytometry for their surface expression. **(C)** THP-1 cells overexpressing different Clec12a mutants as indicated were stimulated with PMA + MSU. 12 h later, IL-1β secretion was detected by ELISA. **(D)** Similar to (C), THP-1 cells overexpressing various Clec12a mutants as indicated were stimulated with PMA + MSU. 12 h later, IL-1β secretion was detected by ELISA. (−4aa indicates XXX, −2aa indicates XXX, +2aa indicates XXX) **(E)** Schematic illustration of leucine(L) to phenylalanine(F) mutations in the Clec12a transmembrane domain, pink highlights indicate L replaced by F. **(F)** as in (B), THP-1 cells with various transfectants of Clec12a mutants (as indicated) were stained with PE-conjugated anti-Clec12a ectodomain antibody and detected by flow cytometry for their surface expression. **(G)** As in (C, D), THP-1 cells overexpressing different Clec12a mutants were stimulated as indicated. 12 h later, IL-1β secretion was detected. Data are represented from of three independent experiments.

C- type lectin family members are single-span type II transmembrane proteins (Dambuza & Brown, 2015). As their TMD are exposed to a high hydrophobic lipid core (Janes et al, 2000; Varshney et al, 2016), a potential mechanism for Clec12a’s inhibitory activity was to use its extracellular C-terminal domain as the specificity determinant for MSU (Neumann et al, 2014), whereas interrupting lipid raft aggregation via its TMD. To address this, the TMD of Clec12 was swapped with three other Clec family members, Clec 4a, 7a, and 9a (Fig 4A). The expression levels were determined to be similar using Clec12a C-terminal-specific antibody (Fig S4A). Flow cytometry analysis further revealed that the mutation of the TMD of CLEC12a does not affect its surface expression or binding affinity to the anti-Clec12a antibody (Fig S4B). Colocalization of Clec12a mutants, lipid rafts, and MSU were imaged as above. Interestingly, swapping with TMDs from Clec7a (TM_Clec7a) or 9a (TM_Clec9a) had results similar to WT Clec12a, yet replacing with Clec4a TMD (TM_Clec4a) significantly diminished the expulsion effect of Clec12 in MSU-induced lipid raft accumulation (Fig 4B and C). Consistently, Western blot results showed that Syk phosphorylation only took place when TMD was replaced by that of Clec4a (Fig 4D). Specifically, TMD switching from Clec12a to Clec4a dampened the inhibitory effect of Clec12a on MSU-induced Syk phosphorylation, indicating the opposite functions of TMDs from Clec4a and Clec12a in transducing MSU-related signals (Fig 4D). To determine the physiological role of the TMD in transducing Clec12a’s inhibitory signal, IL-1β secretion under stimulation was analyzed in various Clec12a TMD mutation transfectants (Fig S4C). Overexpression of WT Clec12a suppressed IL-1β secretion, whereas TMD swapping with Clec4a (but not others) restored the level of secreted IL-1β. To verify this, more precise mutagenesis of TMD in Clec12a was conducted (based on both WT and TM_Clec4a versions of Clec12a) and IL-1β secretion was analyzed. It showed that although elongation or truncation of TMD did not affect the inhibitory effect of WT Clec12a, similar modifications on the TM_Clec4a version of Clec12a, especially four amino acids (aa) deletion in TMD, significantly reduced the secretion of IL-1β, indicating that a mechanism intrinsic to this particular TMD makes it unable to suppress MSU-mediated activation (Fig S4D). Sequencing of TMDs from those Clec family members revealed that 12a, 7a, and 9a are formed mostly by small hydrophobic amino acids in the middle and are expected to form a tightly packed alpha helix in the lipid bilayer (Diaz-Rohrer et al, 2014; Lorent et al, 2017). Clec4a, on the other hand, contains several phenylalanines (Phe or F) which might make the TMD loosely packed as a result of having so many bulky side chains (Diaz-Rohrer et al, 2014; Lorent et al, 2017). To ascertain this, we separately introduced four F amino acids into Clec12a TMD by mutagenesis of Leucine (L) to F (Fig S4E). Surface expressions of various mutants were also ascertained to be equivalent on both THP-1 and Hela cells (Fig S4F). Mutations of leucine to phenylalanine at position 53 and 54 largely impaired membrane targeting of Clec12a (data not shown). For the rest, Clec12a mutants L46F and L57F were less likely to be recruited into and disrupted lipid rafts (Fig 4E), Clec12a-mediated inhibition of MSU induced IL-1β secretion was also removed by F mutations (Fig S4G). As TMD mutations tend to impact surface expression, this work cannot be systematically carried out for all positions; our preliminary data nevertheless indicated that Clec12a may interfere with MSU activation by virtue of a tightly packed alpha helical hydrophobic core of its TMD.

### Clec12a-mediated inhibitory effect is autonomous

**Figure 5.**
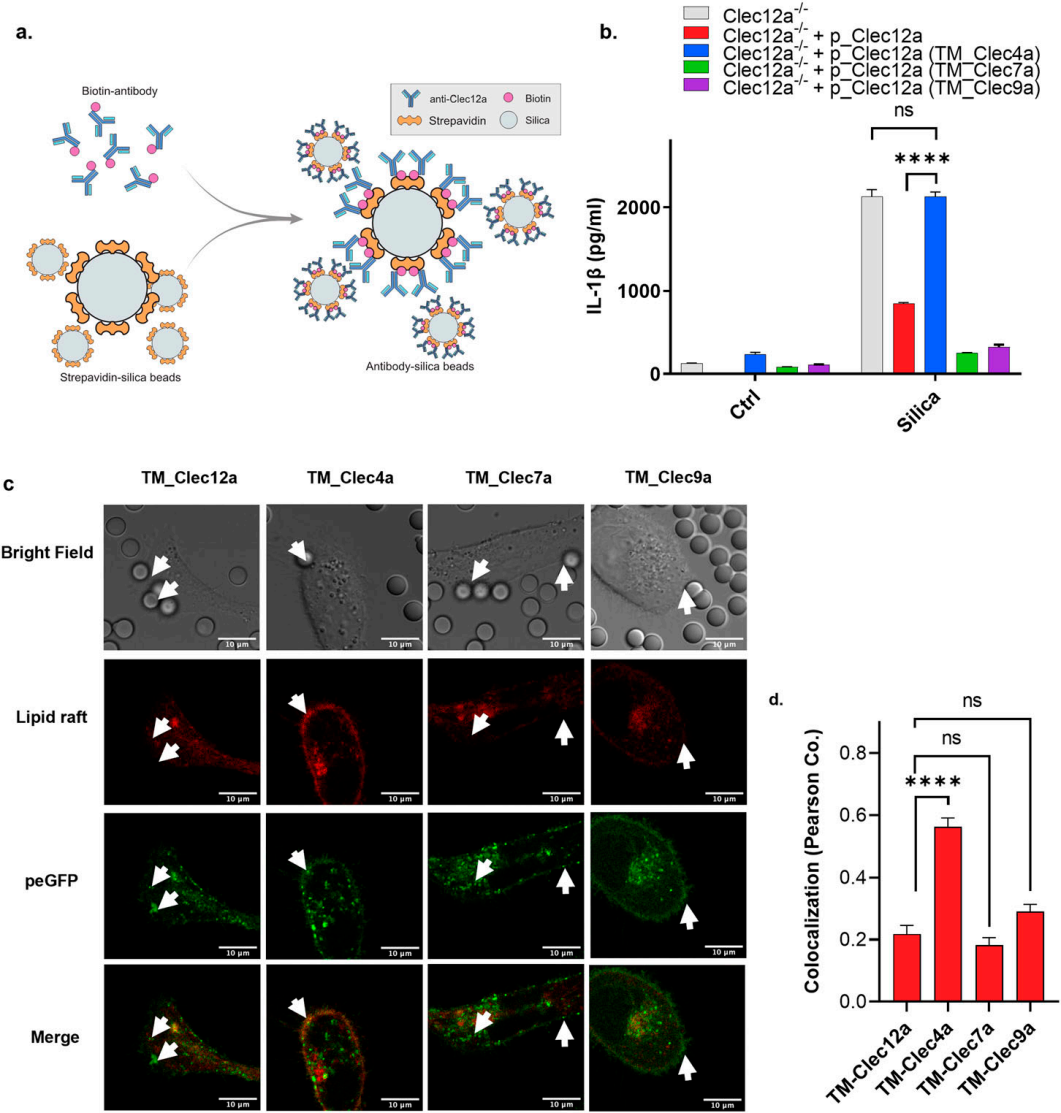
Autonomous effect of Clec12a in disrupting lipid raft aggregation in response to silica stimulation. **(A)** Schematic illustration of silica conjugation and Clec12a recognition. **(B)** THP-1 cells expressed with WT and different transmembrane mutants of Clec12a were stimulated with conjugated silica particles after phorbol 12-myristate 13-acetate (PMA) priming, the secretion of IL-1β was detected by ELISA. **(C)** Confocal imaging of Hela cells overexpressing different transmembrane domain variants of Clec12a and stimulated with conjugated silica particles. All parameters were set as the same as in Fig 3A, bright field images were also captured for silica particle visualization. Arrows indicate MSU-contacted regions on the plasma membrane. Scale bar: 10 μm. **(C, D)** Statistical analysis of the colocalization rate in (C). Data are represented from of three independent experiments.

Our data suggested that Clec12a mediated the inhibitory effect via its TMD, although it remained unknown whether such an effect was applicable in the phagocytosis of other solid crystals. Silica crystals similarly activate phagocytes, whereas no negative receptor has been identified yet (Hornung et al, 2008). To test the inhibitory activity of Clec12a TMD in silica-induced immune activation, streptavidin-coated silica crystals were co-cultured with THP-1 with biotin-labeled Clec12a antibody, which artificially trapped Clec12a underneath the silica crystals on the plasma membrane, creating an artificial colocalization of Clec12a with aggregated lipid rafts formed in response to silica crystal binding (Fig 5A, scheme). IL-1β production was measured accordingly, and it revealed that the TMDs of Clec12a, Clec7a, and Clec12a were all effective in suppressing silica-induced IL-1β secretion. In contrast, TMD replacement with Clec4a essentially removed the inhibitory activity of Clec12a (Fig 5B). Colocalization of different Clec12a mutants with lipid rafts was also visualized and it showed a trend similar to that of MSU with inhibitory TMD-expelling lipid rafts (Fig 5C and D). Taken together, these results revealed that Clec12a TMD operated as a strong interference for crystal-induced lipid raft aggregation, suggesting a new molecular basis for its suppression of MSU-induced immune activation.

### In vivo activity of Clec12a in inhibiting MSU-induced acute inflammation via lipid raft aggregation

**Figure 6.**
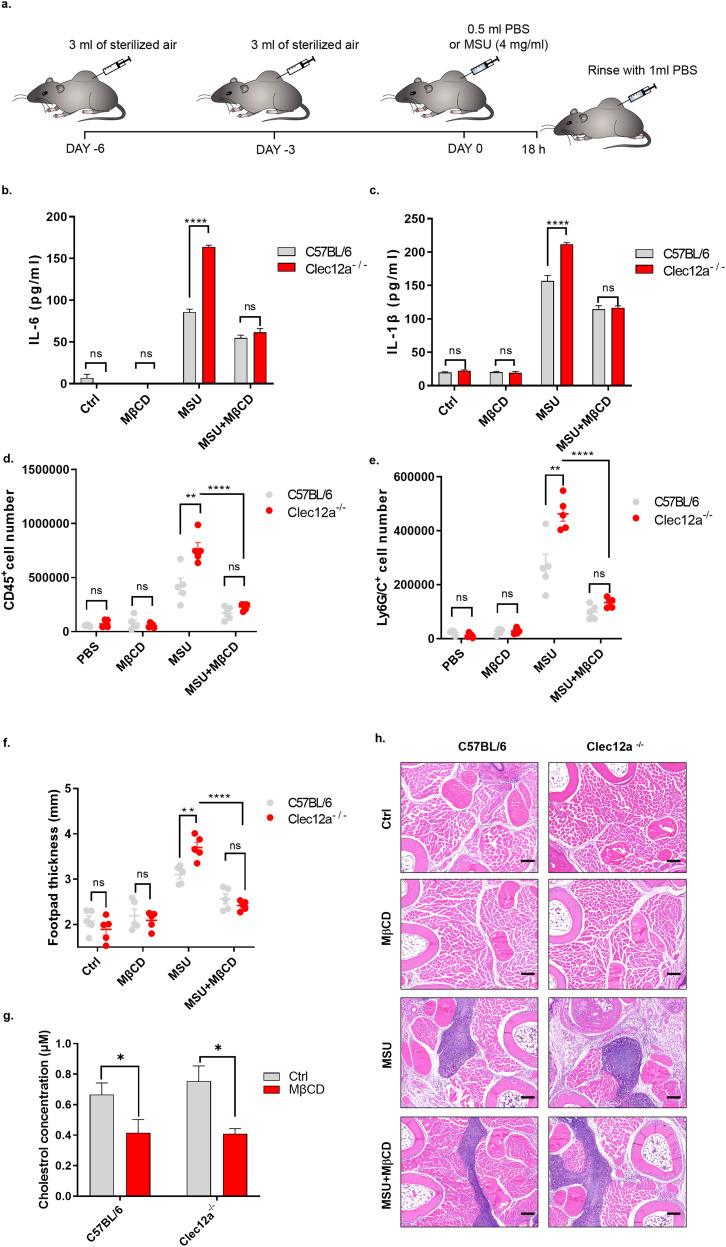
In vivo role of Clec12a in inhibiting monosodium uric acid-induced acute inflammation via lipid raft aggregation. **(A)** Establishment of mouse air pouch gout model via back skin air injection and MSU administration. **(B, C, D)** Fluid in the pouch of mouse back was extracted and the amount of pro-inflammatory cytokines IL-6 (B), and IL-1β (C) in supernatants were detected via ELISA. **(D, E)** Fluid in the pouch of mouse back was extracted and the infiltration of total cells (D) and neutrophils (E) were detected via flow cytometry. **(F)** Thickness of mice footpads under different treatments. **(G)** Efficiency of cholesterol depletion by MβCD in vivo. Infiltrated cells of each group were counted and diluted to 1 million per sample; the concentration of cholesterol was detected as described above. **(H)** H&E staining images of mouse footpads under different treatments as indicated. Magnification: 20x. Scale bar: 100 μm. Five mice per group was setup in mouse experiments and data are represented from of three independent experiments.

**Figure S5.**
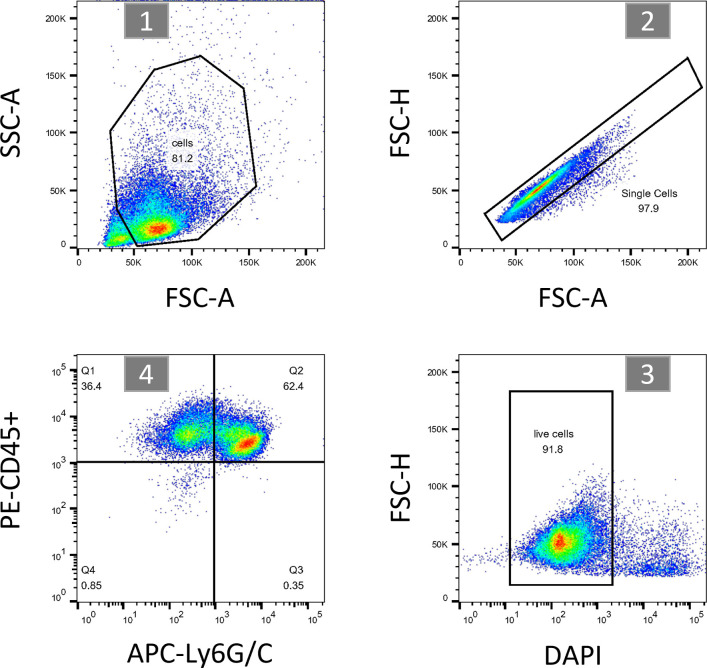
Gating strategy for flow analysis of infiltrated cells in mouse air pouch fluid. Briefly, cells were co-stained with PE-conjugated anti-mouse CD45, APC-conjugated anti-mouse Ly6G/C, and DAPI. The total population of cells were first gated to exclude debris or cell fragments in step 1 (upper left), then single cells were selected in step 2 (upper right), DAPI-negative subpopulation were gated for live cells in step 3 (lower right), total CD45^+^ cells and CD45^+^ Ly6G/C^+^ cells (neutrophils) were displayed (lower left) for statistical analysis in Fig 6D and E.

To better understand the physiological role of Clec12a in MSU-induced inflammation in vivo, a mouse model of air pouch gout was established (Pessler et al, 2008). Briefly, C57BL/6 or *clec12a*^−/−^ mice were injected with sterile-filtered air underneath the back skin, which provided a suitable environment for MSU inoculation and fluid accumulation mimicking the pathogenesis of gout (Fig 6A, scheme). The results showed that MSU injection led to the secretion of proinflammatory cytokines including IL-1β and IL-6 in both C57BL/6 and *clec12a*^−/−^ mice, whereas the amount of secreted cytokine in the *clec12a*^−/−^ group was higher compared with WT control. Meanwhile, dual administration of MSU and MβCD led to a significant abrogation of cytokine secretion in both groups, and the fold difference was no longer found between C57BL/6 and *clec12a*^−/−^ mice. Sole administration of MβCD did not induce any inflammation as expected (Fig 6B and C). These results suggest that the inhibitory effect of Clec12a was no longer applicable in the absence of lipid rafts. Flow cytometry was performed for neutrophil infiltration analysis (Fig S5 for gating strategy). It revealed that the infiltration of neutrophils upon MSU administration and the number of infiltrated cells in *clec12a*^−/−^ mice were significantly higher than the one in WT control. However, MβCD co-treatment suppressed the infiltration of neutrophils in responses to MSU and no difference was observed between C57BL/6 and *clec12a*^−/−^ mice (Fig 6D and E). To verify this, we established another mouse model via intra-plantar injection of MSU crystals (Lin et al, 2020; Shin et al, 2020). 18 h later, foot swelling of mice under different treatments was compared and inflammatory responses were evaluated. It showed that MSU administration induced more severe foot swelling in *clec12a*^−/−^ mice than WT control, which was significantly relieved when treated in combination with MβCD (Fig 6F). The amount of retained cholesterol on infiltrated cells of each group was quantified, it showed that MβCD injection led to the reduction of cholesterol on cells (Fig 6G). Histopathologic analysis also revealed the differential infiltration of lymphocytes and was attenuated by co-administration of MβCD (Fig 6H). Collectively, these results demonstrated the physiological role of Clec12 in MSU-induced immune activation in vivo, which highlight the importance of lipid raft in transducing Clec12a-initiated signals in response to MSU stimulation.

### Computational analysis of Clec12a TMD in interrupting MSU-induced lipid reorganization

**Figure 7.**
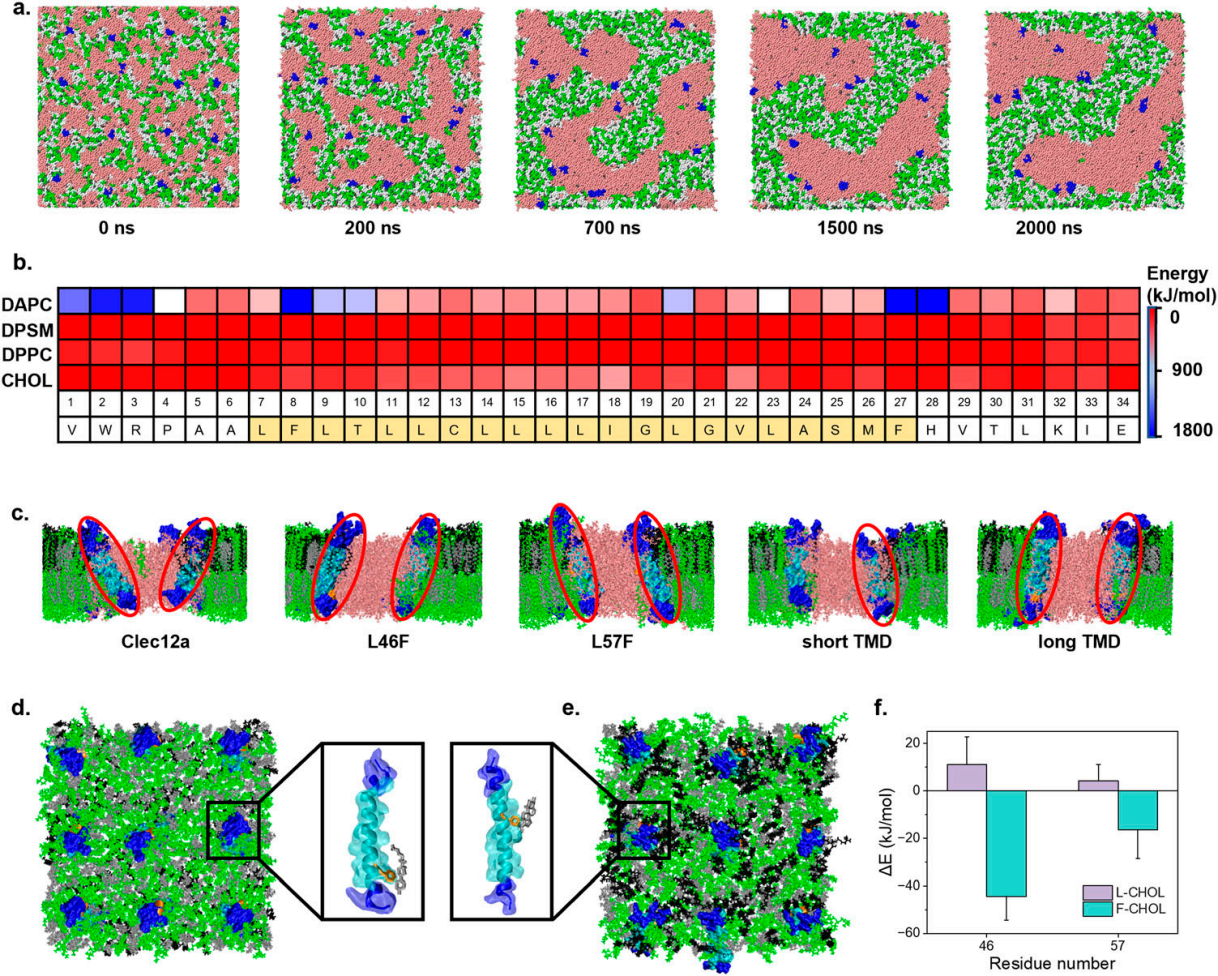
Molecular dynamics simulations of Clec12a interactions with lipid domains. **(A)** CG MD time sequence of typical snapshots depicting WT Clec12a TMD partitioning to the Ld domain. Proteins are displayed in blue, unsaturated lipids are displayed in pink, whereas saturated lipids are green. Water molecules are not displayed for clarity. **(B)** Energies of interaction between each amino acid with different lipid components in the membrane. **(C)** AA MD-simulated snapshots of different Clec12a mutants with a phase-separated membrane. **(D)** L46F mutant interaction with a pure Lo membrane, the right panel is an enlarged structure showing π-π stacking between the side chain of benzene in F46 and surrounding cholesterol. **(E)** L57F mutant interaction with a pure Lo membrane, the left panel shows π-π stacking between side chain of benzene in F57 and surrounding cholesterol. **(F)** Change of interaction energy between specific residues and cholesterol. TMD, transmembrane domain; DAPC, unsaturated 1,2-diarachidonoyl-sn-glycero-3-phosphocholine DPSM, sphingomyelin; DPPC, 1,2-dipalmitoyl-sn-glycero-3-phosphocholine; CHOL, cholesterol; CG, coarse-grained; AA, all atom; MD, molecular dynamic; Lo, liquid ordered; Ld, liquid disordered.

In general membrane biology, biophysics analysis tends to suggest that receptor–ligand ligation leads to the demobilization of receptors, indicating a state of activation. In the case of Clec12a, we wondered if the high aggregation of Clec12a TMD can also block lipid domain formation. To address this, coarse-grained (CG) molecular dynamics (MD) simulations were performed on TMD of WT Clec12a in a multi-component membrane experiencing phase separation (Monticelli et al, 2008; Wu et al, 2014). Simulation results showed that most of the TMD of WT Clec12a were partitioned to the boundaries between Lo domain and Ld domain (Fig 7A), and showed higher affinity to unsaturated 1,2-diarachidonoyl-sn-glycero-3-phosphocholine (DAPC) in the disordered domain (Fig 7B), which might contribute to the ability of Clec12a to pull MSU out of lipid rafts because Clec12a can switch between Lo and Ld domains. To understand how different mutations influence Clec12a partitioning in the phase-separated membrane, all-atom (AA) MD simulations were performed. Compared with TMD of WT Clec12a, L46F and L57F mutants were more likely to associate with the Lo domain (lipid raft, Fig 7C), which is consistent with confocal imaging results and the limited inhibitory effect of swapped Clec4a TMD in response to MSU. To elucidate why such mutation favors lipid rafts, AA MD simulations were performed on L46F and L57F mutants of Clec12a in the Lo domain. Interestingly, the side chain of benzene in F46 and F57 formed π-π stacking with surrounding cholesterol (Fig 7D and E). We calculated change of the interaction energy between specific residues and cholesterol (Fig 7F). The positive values for L46 and L57 manifested unfavorable association between WT Clec12a and lipid raft. After mutation, the energy changes became negative, suggesting favorable interactions of mutants with lipid raft. By contrast, L46F induced lower energy than L57F because the residue of F46 locates shallower in the membrane to form more stacking contacts with cholesterol. Collectively, these results suggested a potential molecular mechanism of Clec12a TMD in inhibiting MSU-induced inflammatory signals via interference of lipid sorting.

## Discussion

Immune sensing of solid particle is of interest in the field of innate immunity because of their adjuvanticity and their ability to drive crystallopathy. However, the underlying mechanism of solid particle recognition by immune cells has not been clarified yet, possibly because of the diversity and complexity of their molecular structures (Shi, 2012). As one of the most well-established DAMPs, MSU crystal was identified as the causative agent of gout (Shi et al, 2003; Rock et al, 2013) and has been extensively studied for its role in inflammation and innate immune response. It was shown that MSU has a strong ability to drive inflammation via NLRP3 inflammasome activation (Martinon et al, 2006) and Syk-dependent inflammatory phagocytosis (Ng et al, 2008). Whether there is a cell-surface receptor for MSU had remained a mystery. Neumann et al identified Clec12a as the receptor for MSU on the plasma membrane by constructing the fusing protein with the extracellular domain of Clec12a and Fc part of IgG, the recognition of MSU by Clec12a was also revealed by the cell reporter system (Neumann et al, 2014). Although the interaction between Clec12a and MSU was established, it is still unclear about the mechanism for Clec12a’s inhibitory function. ITIM motif in Clec12a per current dogma appears to be sufficient in explaining the attenuation. This proposal had not been vigorously tested. Our current study shows that Clec12a interrupts MSU-induced lipid raft aggregation to produce the inhibitory effect. This finding illustrates how this protein receptor Clec12a can negatively impact a lipid sorting-based innate activation. We, for the moment, do not know why the ITIM signaling, which is intact in our system by co-precipitation assay, did not contribute to the inhibition. One possibility is that the membrane lipid ligation-mediated phagocytosis is exceedingly strong and rapid, and the dephosphorylation event may be of limited potency in reversing the process. This possibility remains to be tested in the future.

Regarding the molecular mechanism of Clec12a interfering with MSU-induced lipid sorting, our molecular simulation data suggested that the TMD of WT Clec12a has more favorable interactions with the Ld domain. Given this fact, upon Clec12a binding with MSU, it inhibits the recruitment of Lo domain in the MSU-contacted region. L to F mutations on position 46 or 57 promotes the interaction of Clec12a to the Lo domain, which reduced the threshold for MSU-induced lipid sorting in the presence of Clec12a. Previous studies proposed key determinants of the transmembrane sequence in interacting with lipid domains, which include the length, amino acid composition, modifications, etc. (Lorent et al, 2017). Our simulated data suggested that differential behaviors of Clec12a and its mutants in interacting with the cell membrane are subjected to enthalpy-driven partitioning of Clec12a in different lipid domains. Because the Lo phase is relatively tight, and interactions between surrounding lipids are strong, introduction of external proteins may cause a huge loss of enthalpy, which may not be offset by the increase in entropy during the process. The Ld phase, on the other hand, is less dense and more favorable for Clec12a, and because lipids in the Ld phase are already poorly organized, introduction of protein does not lead to significant increases of entropy. Therefore, Clec12a repels the Lo phase whereas having stronger affinity to the Ld phase. The fact that L-F mutation drives Clec12a toward the Lo phase can be explained by an additional finding: the π-π interaction of F residues in the TMD with cholesterol in Lo. It is likely that both mechanisms are at work to promote better acceptance of non-inhibiting, Clec4a-like TMD into lipid rafts. In contrast, forced entry of an inhibiting TMD, such as under the pulling force of extracellular Clec12a binding to MSU surface, disrupts lipid sorting and the ensuing phagocytic signaling. In addition, it was reported that phosphorylation of the Clec12a ITIM domain depends on receptor clustering in flotillin-rich membrane domains and this could be disrupted by MβCD (Paré et al, 2021). Therefore, it would be intriguing to compare the phosphorylation of SHP-1/2 of various Clec12a TMD mutants, which would be insightful for future studies on the relationship between lipid membrane partitioning of receptors and intracellular signal transduction.

Despite continuous debates for decades, the concept of lipid raft, which refers to microdomains enriched in saturated lipids, sphingolipids, and cholesterol, on the plasma membrane, has been well accepted by communities as the “supporting platform” where most membrane receptors can be activated (Hancock, 2006). Biophysical studies revealed that, in contrast to Ld phases containing loose-packed unsaturated lipid species, the physical chemistry of lipid raft (or Lo domain) is more rigid because of tight interactions among different components, which supports the conformational and oligomerizing state alteration of membrane proteins required for signal transduction (Dietrich et al, 2001; Kaiser et al, 2009). The molecular basis for lipid raft formation is the evident affinity between cholesterol and saturated lipids, whereas both of them are repulsive to unsaturated lipids (Levental et al, 2020), which ultimately lead to liquid–liquid phase separations (Dietrich et al, 2001). In living cells, lipid rafts are more dynamic because they are regulated by variable factors, of which, transmembrane proteins are in general detrimental for the formation and dissociation of lipid rafts through peptide–lipid interactions (Simons & Sampaio, 2011; Sezgin et al, 2017). It has been proposed that several transmembrane protein motifs are responsible for specific interactions with different lipid species. For instance, Pleckstrin homology (PH) domains and Phox homology (PX) domains are predominantly responsible for recognizing phospholipids; the cholesterol consensus motifs (characterized by L/V(X)(1–5)Y(X)(1–5)R/K) are responsible for cholesterol recognition; whereas V(XX)TL(XX)IY is one of the best characterized sphingolipid-binding motifs (Björkholm et al, 2014). Nevertheless, a universal rule for lipid–peptide interaction is still lacking because of insufficient information by far. Our data suggested that, in the context of Clec12a, single amino acid mutation (L-F) in the TMD is sufficient to permit lipid domain formation, which appears to be less stringent than other lipid recognition motifs. One unexpected result is that three out of four Clec family members tested show a similar ability to block MSU-mediated activation, with the only exception of Clec4a with a total of six F in its TMD. Whether type II TMDs with packed transmembrane helix all have similar ability to interfere with lipid raft organization when co-localized is unknown. If so, whether addition of large side chain aa reverses the inhibition is also intriguing. The inhibitory effects of Clec12a exerted by its TMD are conceptually novel. Although how often this mechanism in used in type II transmembrane proteins remains a new territory, our work may help pave the way for developing membrane–lipid-targeted drugs/therapies, such as in the clinical treatment of gout.

There are several limitations of our study. First, it remains elusive for the involvement of membrane lipids in the recognition of Clec12a and MSU crystals. A previous study has shown that MSU specifically binds to Clec12a-Fc fusion protein but not Clec7a-Fc or Clec9a-Fc, indicating the direct interaction between Clec12a and MSU (Neumann et al, 2014). However, it was performed in a cell-free system that the effect of lipid membrane, particularly lipid rafts, in MSU recognition by Clec12a was not addressed. The binding activity of Clec4a with MSU and the role of lipid raft in such a process have not been determined as well. Regarding the imaging analysis, it was reported that the CTB pentameric structure can bind five molecules of GM1 and induce coalescence of lipid rafts (Day & Kenworthy, 2015). Although we have performed GP analysis to verify the formation of lipid raft under the MSU-contacted region, additional visualizing approaches are still required to further validate the involvement of Clec12a in the dynamics of membrane lipids in the formation of lipid rafts. Another limitation is the lack of strategies for investigating the biological functions and underlying mechanism of lipid rafts in vivo. Unlike proteins that can be precisely manipulated with genetical and chemical interventions, there are very few approaches for lipid raft manipulation. MβCD has been widely used for lipid raft disruption based on its capability of cholesterol extraction, although it could lead to other unexpected consequences. These questions would need to be investigated by follow-up studies in the future.

## Materials and Methods

### Mice

C57BL/6 mice were obtained from Jackson Laboratories. *clec12a*^−/−^ mice were kindly provided by Dr. Xin Lin’s laboratory from School of Medicine, Tsinghua University. All mice were bred and housed per approved protocols at Tsinghua University Animal Facilities (ICUAC).

### Cell culture

Hela cells were kindly provided by Dr. Xiaohong Fang’s laboratory from the Institute of Chemistry, China Academy of Science, and 293FT cells were kindly provided by Dr. Yeguang Chen’s laboratory from the School of Medicine, Tsinghua University, DC2.4 and THP-1 were kindly provided by Dr. Kenneth Rock’s laboratory from the University of Massachusetts Medical School, THP-1 and Raw264.7 cells were obtained from ATCC. Cell culture follows ATCC instructions that all immune cell types like DC2.4, THP-1, and Raw264.7 were cultured in RPMI-1640 medium supplemented with 10% FBS, 100 U/ml penicillin/streptomycin, 10 mM HEPES, and 50 μM β-mercaptoethanol complete culture medium (CCM), non-immune cell types like Hela and 293FT were cultured in DMEM supplemented with 10% FBS and 100 U/ml penicillin/streptomycin. Murine BMDC were induced from mouse bone marrow cells following the protocol of a previous publication (Son et al, 2002). Briefly, RBC-lysed mouse bone marrow cells were cultured in CCM with 20 ng/ml GM-CSF and 10 ng/ml IL-4, the medium was half-refreshed after 3 d, BMDC was obtained after 7 d of culture and used immediately in the following experiments. Differentiation of THP-1 cell was modified from previous reports (Daigneault et al, 2010). Briefly, the floating, monocyte-like THP-1 cells were stimulated with 10 ng/ml Phorbol 12-myristate 13-acetate (PMA) in CCM for 48 h until transformed to an adherent, macrophage-like type for the following experiments.

### Plasmids

pLVX-IRES-zsGreen plasmid was kindly provided by Dr. Wei Guo’s laboratory from School of Medicine, Tsinghua University. pCAGin and pCAGin-N-EGFP plasmids were customized by our laboratory, lentiCRISPR v2 (Plasmid #52961) was obtained from Addgene. All plasmids were verified by sequencing before use.

### Reagents

Murine GM-CSF (315-03-1000) and murine IL-4 (214-14-000) were obtained from Peprotech; protein A/G agarose was obtained from Abmart; sodium orthovanada was obtained from Yeason Biotech; phorbol 12-myristate 13-acetate (PMA)/12-O-tetradecanoylphorbol 13-acetate (TPA) mixture solution (S1819) and PMSF were obtained from Beyotime biotech; Lipofectamine 2000 (11668019) was obtained from Thermo Fisher Scientific; β-mercaptoethanol (07604) and DMSO (V900090) were obtained from Sigma-Aldrich. Biotin Labeling Kit (E-LK-B002) was obtained from Elabscience. Di-4-ANEPPDHQ (D36802) was from Thermo Fisher Scientific. Total cholesterol (TC) content assay kit (BC1985) was obtained from Solarbio. Alexa Flour 647-conjugated Annexin-5/PI kit was purchased from 4A biotech.

### Antibodies

Anti-human IL-1β functional grade purified antibody (16-7018-85), anti-IL-1beta monoclonal antibody (13-7016-85), anti-mouse IL-1beta capture antibody were obtained from eBioscience; anti-Clec12a (C-terminal) (ab193941) was obtained from Abcam; HRP-conjugated anti-mouse IgG (7076), β-actin (13 × 10^5^) rabbit mAb (4970) and anti-human Caspase-1 were obtained from CST; anti-mouse Caspase-1 p20 (AG-20B-0042) was obtained from Adipogen; human MICL/Clec12a antibody (MAB29461-100) and mouse MICL/Clec12a antibody (AF2950) were obtained from R&D systems; PE-conjugated rat anti-mouse CD371 (Clec12a) antibody (562773) was obtained from BD biosciences; PE conjugated anti-human CD371 (Clec12a) monoclonal antibody (HB3) (12-9878-42), allophycocyanin-conjugated p-Syk (Tyr348) monoclonal antibody (mono1ct) (17-9014-42), fluorescein isothiocyanate-conjugated goat anti-rabbit IgG F(ab′)2 secondary antibody (31573), and fluorescein isothiocyanate-conjugated rabbit anti-goat IgG F(ab′)2 secondary antibody (31553) were obtained from Invitrogen; HA-tag (26D11) mouse monoclonal antibody (M20003L) was obtained from Abmart; mouse IgG1 isotype control antibody (130-113-762) was obtained from MACS.

### MSU crystal preparation

The preparation of crude MSU crystals (20–50 μm long in needle shape) was done following the published report (Luo et al, 2020). In brief, 1.68 g uric acid (Sigma-Aldrich) was dissolved in 400 ml of 25 mM NaOH solution and then left for overnight at RT. Crystals were harvested the next day after decanting off the supernatant. After washing three times with cold sterile PBS and filtrated on a filter paper, crystals were left to dry completely at RT in a hood with fan for 3 d. Crude MSU was further placed into a 50 ml tube with appropriate amount of steel beads and homogenized for 6-h to make fine MSU (1–3 μm in diameter).

### Molecular cloning

Human *clec12a* gene was cloned from the cDNA library of THP-1 and mouse *clec12a* gene was cloned from the cDNA library of bone marrow cells from C57BL/6 mice. The primer sequences are as follows: (5′–3′): murine *clec12a* forward, ATGTCTGAAGAAATTGTTTA; murine *clec12a* reverse, CTACCTGCTATCCTCTGGGAGGCCA; human *clec12a* forward, ATGTGGATAGATTTCTTTAC; human *clec12a* reverse, TCATGCCTCCCTAAAATATG. All mutageneses of WT human or mouse Clec12a are detailed in the text. PCR-amplified fragments were cloned into pCAGin-N-EGFP or pLVX-zsGreen, respectively, and all constructs were validated by sequencing.

### Flow cytometry

For cell surface staining and FACS analysis, cells were stained with the indicated fluorophore-conjugated antibodies for 15 min under dark at RT, and then samples were washed with FACS buffer (PBS with 2 mM EDTA) three times before flow analysis. For p-Syk flow analysis, cells were fixed, permeabilized, and stained with fluorophore-conjugated anti-p-Syk antibody for 1 h under dark at RT. Then, the samples were washed three times before FACS analysis. Fluorescent intensity of 1 × 10^3^ was set as the cut-off value for p-Syk phosphorylation as previously described (Ng et al, 2008). For cell death detection, cells were treated with 10 mM MβCD or left untreated for 1 h, then the cells were washed with pre-warmed PBS for three times and incubated for an additional 5 h. The samples were stained with Annexin-5/PI according to manufacturer’s protocol and cell death events were recorded by flow cytometry.

### Cell transfection

For transient transfection, cells were cultured in six-well plates until 70% confluent. 4 μg of endotoxin-free plasmids and 10 μl Lipofectamine 2000 were dissolved in 250 μl Opti-MEM for 5 min, respectively, then mixed and stayed for another 20 min before dropping into each well. 6 h later, cell supernatants were discarded and replaced with a fresh medium. 36–48 h later, transfection efficiency was monitored by fluorescent imaging (for vectors with fluorescent tag) or drug screening (for vectors with drug-resistant tag). To obtain chromosome-integrated transfectants, cells were treated with 250 ng/ml G418 screening and single clones were picked up by serial dilution. Overexpression of the inserted genes was verified by FACS or Western blot analysis. For lentiviral-mediated transfection, 293FT cells were transfected with lentiviral vectors along with the packaging plasmids pMD2.G and psPAX2 following the weight ratio of 4: 1: 3. To transfect one well in a six-well plate, the total amount of 9 μg plasmids and 9 μl of Neofect was used for making the mixture and the procedure is similar to transient transfections. 48–72 h later, cell supernatants were collected and filtered through a 0.45-μm syringe filter. For lentiviral infection of THP-1 cells, cells were incubated with a lentiviral supernatant and 8 μg/ml polybrene and centrifuged at 3,000*g* for 30 min; later, the cell pellets were resuspended in CCM with 1 μg/ml puromycin for selection. Positive clones were picked up, amplified, and verified by Western blot analysis.

### Cell assay

For MSU stimulation, cells were treated with 100 μg/ml fine MSU crystals for indicated time or otherwise indicated; for MβCD treatment, cells were treated with 10 mM MβCD 1 h before additional stimulations. To detect Syk phosphorylation, 1 mM Na_3_VO_4_ was added into cell cultures for 20–30 min before analysis.

For cholesterol quantification, cells were washes with PBS for three times and the remaining cholesterol on cells of each group was detected via the total cholesterol quantification kit according to the manufacturer’s protocol. Briefly, cells were broken by an ultrasonic wave on ice (power 300 W, ultrasonic 2 s, interval 3 s, total time 3 min), centrifuged at 10,000*g* 4°C for 10 min, and then placed for test. For cholesterol quantification of cells collected from air-pouch fluid, cells from each sample were first counted and diluted to equal number (one million per sample) before detection to preclude the influence cell number on final readouts.

### Real-time PCR

For real-time PCR analysis, total amount of RNA was extracted by Trizol and retro-transcribed into cDNA. The following primers were used to detected the expression of human or mouse CD80/86: human *cd80* Forward: AAACTCGCATCTACTGGCAAA; human *cd80* Reverse: GGTTCTTGTACTCGGGCCATA; human CD86 Forward: CTGCTCATCTATACACGGTTACC; human *cd86* Reverse: GGAAACGTCGTACAGTTCTGTG; mouse *cd80* Forward: CTGCAAAGGACTTCAGAAACCT; mouse CD80 Reverse: AGGCTTCACCTAGAGAACCGT; mouse *cd86* Forward: GGTGGCCTTTTTGACACTCTC; mouse *cd86* Reverse: TGAGGTAGAGGTAGGAGGATCTT; GAPDH Forward: ATCAAGAAGGTGGTGAAGCA; GAPDH Reverse: AGACAACCTGGTCCTCAGTGT.

### Co-IP

For SHP-1/2 immunoprecipitation, 4 μg anti-HA antibody were incubated with 100 μl protein A/G agarose for 2 h at RT. Cells (3 million/ml) were collected and lysed with immunoprecipitation lysis buffer (50 mM Hepes, pH 7.4, 150 mM NaCl, 1% NP-40, 1 mM EDTA, 1 mM PMSF, 1 mM NaF, 1 mM NaVO3, and protease inhibitor cocktails). The cell lysates were added into antibody–bead mixtures for 3 h at 4°C. Beads were then collected by low-speed centrifugation (1,000*g* for 5 min) and washed four times with immunoprecipitation lysis buffer (300 mM NaCl added). After the last wash, pellets were suspended with 70 μl 2× SDS loading buffer and boiled for 5 min before Western blot analysis.

### Confocal microscopy

For endocytic efficiency detection, latex beads were mixed with 0.1 M borate buffer (pH 8.4), centrifuged at 1,500*g* for 5 min, and washed with 0.1 M borate buffer for an additional two times. Later, the latex beads were mixed with biotin-BSA at the final concentration of 350 μg/ml and incubated overnight, then washed with 1x PBS two times, and centrifuged at 1,500*g* for 5 min. The coated beads were stored at 4°C before use.

For lipid raft and Clec12a visualization under MSU stimulation, endogenous Clec12a-deficient Hela cells overexpressing indicated Clec12a-EGFP mutants were stained with CTB-647 at 37°C for 15 min, then monitored under ZEISS LSM 880 confocal laser scanning microscope. Images were captured and processed with Image J. For colocalization analysis between lipid raft and Clec12a, MSU-contacted regions (Regions of Interest) were picked up and the colocalization rate (Pearson’s correlation coefficient) between green signal (Clec12a) and red signal (lipid raft) were calculated basing on the fluorescent intensities via Image J plugin JACoP_.jar (2.1.1) (Bolte and Cordelières, 2006). For colocalization analysis between MSU and lipid raft, the number of “CTB positive, MSU contacted” regions on cell membranes of each image was counted and divided by the total MSU-contacted regions in the same image. A total of 15 images of each group were processed for statistical analysis.

For silica stimulation and confocal imaging analysis, anti-Clec12a antibody was conjugated with biotin molecule following manufacturer’s instructions. Later, endogenous Clec12a-deficient Hela cells overexpressing indicated Clec12a-EGFP mutants were incubated with biotin-conjugated anti-Clec12a Ab and streptavidin-conjugated silica crystals for confocal imaging. Image J was used to analysis the rate of colocalization under different conditions as above.

### GP imaging

Di-4-ANEPPDHQ is a potentiometric styryl dye that carries a dipole sensitive to lateral lipid packing density. Different densities of lipid domains are associated with “relaxed” or “rigid” states. Di-4-ANEPPDHQ is excited at 488 nm and the peak of its emission wavelength is subjected to the polarizing state of the surrounding membrane. In the Lo phase, the emission wavelength of di-4-ANEPPDHQ peaked at 550 nm, whereas it shifted to around 650 nm in the Ld phase (Jin et al, 2006). The generalized polarization (GP) analysis is a digitizing process to convert the fluorescein signal within a given range to a numeric value between −1 to 1. This derivatization produces a new image indicating intensity change between the original set boundaries. As previously described (Song et al, 2019), cells were stained with 5 μM Di-4-ANEPPDHQ in a serum-free medium for 30 min and washed three times with PBS. Samples were monitored under ZEISS LSM 880 confocal laser scanning microscope excited at 488 nm. Fluorescent signals of channel 1 (Ch1: 550–570 nm) and channel 2 (Ch2: 660–560 nm) were recorded. For generalized polarization (GP) analysis, the GP value is calculated basing on fluorescent intensity from each channel according to the following formula: GP=(Ch1-Ch2)/(Ch1+Ch2). Pseudo-color images were generated via Image J plugin (Calculate_GP_2). All images were processed via Image J.

### Air pouch and MSU injection-induced gout model

The mouse model of air pouch-induced goat was established as described previously. Briefly, mice were injected twice with 3 ml of sterilized air under the back skin in 3-d interval. Later, 400 μl of MSU crystals (5 mg/ml in PBS) or equal volume of PBS were injected into the air pouch to induce acute inflammation; for certain groups, MβCD (0.35 M, 4 μl/mice) was injected 20 min later. 18 h later, air pouches were rinsed with 1 ml PBS and extracted fluids were centrifuged. The supernatants and cell pellets were used for ELISA and flow analysis, respectively.

### MSU intra-plantar injection induced gout model

40 μl MSU crystals (50 mg/ml in PBS) or PBS were intra-plantar-injected into mice; for certain groups, MβCD (0.35 M, 4 μl/mice) was injected 20 min later. 18 h later, mice were euthanized and foot swelling was compared. The inflammation of mice feet under different treatments was analyzed by H&E staining and histopathological analysis.

### Hematoxylin and eosin staining

Footpad samples obtained 18 h after MSU crystal injection were immediately fixed in 4% buffered formalin for 48 h at RT. Formalin-fixed paraffin sections (4-μm thickness) were stained using hematoxylin and eosin. The tissue sections were deparaffinized and treated using 3% hydrogen peroxide in methanol to eliminate endogenous peroxidase activity before H&E staining. The resulting images were examined using light microscopy (Olympus).

### MD simulation

To extend time and length scales to be appropriate for simulating membrane phase separation and Clec12a TMD portioning, the widely used Martini CG force field was adopted (Monticelli et al, 2008). The multi-component membrane of 37 × 37 nm in lateral size was prepared using the CHARMM-GUI tool (Wu et al, 2014). Based on the ternary model consisting of saturated 1,2-dipalmitoyl-sn-glycero-3-phosphocholine (DPPC), unsaturated 1,2-diarachidonoyl-sn-glycero-3-phosphocholine (DAPC), and cholesterol (CHOL), asymmetric lipid distributions across two leaflets were considered by introducing sphingomyelin (DPSM) to the outer leaflet. The lipid composition ratio in the outer leaflet was DPPC: CHOL: DPSM: DAPC = 2:2:3:3, and the ratio in the inner leaflet was DPPC: CHOL: DAPC = 3:3:4. AA models of the WT Clec12a were derived from the AlphaFold Data Bank, with the TMD truncated for use in simulations. The AA protein model was transferred to the corresponding CG models using the martinize.py script provided by the Martini force field. CG MD simulations were performed with the NPT ensemble using the semi-isotropic Berendsen barostat (*P* = 1 bar) with a coupling constant of 4 ps and compressibility of 5 × 10^−5^ bar^−1^ in both lateral and membrane normal directions. The temperature was kept constant at T = 295 K using the V-rescale thermostat with a coupling constant of 4 ps. A cutoff of 1.2 nm was used for van der Waals interactions, and the Lennard–Jones potential was smoothly shifted to zero between 0.9 and 1.2 nm to reduce the cutoff noise. The coulombic potential, with a cutoff of 1.2 nm, was smoothly shifted to zero from 0 to 1.2 nm. The particle-mesh Ewald summation method was used to treat the long-range electrostatic interactions. The time step of simulations was 10 fs, with the neighbor list updated every 10 steps.

To acquire atomic-level information about how specific mutations influence Clec12a TMD interactions with lipid raft, a phase-separated membrane of 11.5 × 11.5 nm of the same composition ratio was constructed. Besides, a pure liquid-ordered (Lo) membrane consisting of DPPC, DPSM, and CHOL was also constructed to investigate interactions of Clec12a mutants with lipid raft. Periodic boundary conditions were considered in all three directions. Both CG and AA MD simulations were performed using the GROMACS 2019 software package (Rakhshani et al, 2019). Simulated snapshots were rendered by VMD (Humphrey et al, 1996).

### Statistics

All experiments were repeated independently for at least three times. For mouse experiments, five mice per group were included. All plot graphs show the mean ± SEM. Statistical analysis for each independent experiment was performed with an unpaired *t* test. *P*-value of <0.05 was considered significant: *P < 0.05; **P < 0.01; ***P < 0.001; NS, not significant.

## Data Availability

The data that support the findings of this study are available from the corresponding author upon request.

## Supplementary Material

Reviewer comments

## References

[bib1] Björkholm P, Ernst AM, Hacke M, Wieland F, Brügger B, von Heijne G (2014) Identification of novel sphingolipid-binding motifs in mammalian membrane proteins. Biochim Biophys Acta 1838: 2066–2070. 10.1016/j.bbamem.2014.04.026 24796501

[bib2] Bolte S, Cordelières FP (2006) A guided tour into subcellular colocalization analysis in light microscopy. J Microsc 224: 213–232. 10.1111/j.1365-2818.2006.01706.x 17210054

[bib3] Carquin M, Pollet H, Veiga-da-Cunha M, Cominelli A, Van Der Smissen P, N’Kuli F, Emonard H, Henriet P, Mizuno H, Courtoy PJ, (2014) Endogenous sphingomyelin segregates into submicrometric domains in the living erythrocyte membrane. J Lipid Res 55: 1331–1342. 10.1194/jlr.m048538 24826836 PMC4076090

[bib4] Chen C-J, Shi Y, Hearn A, Fitzgerald K, Golenbock D, Reed G, Akira S, Rock KL (2006) MyD88-dependent IL-1 receptor signaling is essential for gouty inflammation stimulated by monosodium urate crystals. J Clin Invest 116: 2262–2271. 10.1172/jci28075 16886064 PMC1523415

[bib5] Corradi V, Mendez-Villuendas E, Ingólfsson HI, Gu R-X, Siuda I, Melo MN, Moussatova A, DeGagné LJ, Sejdiu BI, Singh G, (2018) Lipid–protein interactions are unique fingerprints for membrane proteins. ACS Cent Sci 4: 709–717. 10.1021/acscentsci.8b00143 29974066 PMC6028153

[bib6] Daigneault M, Preston JA, Marriott HM, Whyte MKB, Dockrell DH (2010) The identification of markers of macrophage differentiation in PMA-stimulated THP-1 cells and monocyte-derived macrophages. PLoS One 5: e8668. 10.1371/journal.pone.0008668 20084270 PMC2800192

[bib7] Dambuza IM, Brown GD (2015) C-type lectins in immunity: Recent developments. Curr Opin Immunol 32: 21–27. 10.1016/j.coi.2014.12.002 25553393 PMC4589735

[bib8] Day CA, Kenworthy AK (2015) Functions of cholera toxin B-subunit as a raft cross-linker. Essays Biochem 57: 135–145. 10.1042/bse0570135 25658350 PMC4346142

[bib9] Devaux PF (1992) Protein involvement in transmembrane lipid asymmetry. Annu Rev Biophys Biomol Struct 21: 417–439. 10.1146/annurev.bb.21.060192.002221 1525472

[bib10] Diaz-Rohrer BB, Levental KR, Simons K, Levental I (2014) Membrane raft association is a determinant of plasma membrane localization. Proc Natl Acad Sci U S A 111: 8500–8505. 10.1073/pnas.1404582111 24912166 PMC4060687

[bib11] Dietrich C, Bagatolli LA, Volovyk ZN, Thompson NL, Levi M, Jacobson K, Gratton E (2001) Lipid rafts reconstituted in model membranes. Biophys J 80: 1417–1428. 10.1016/s0006-3495(01)76114-0 11222302 PMC1301333

[bib12] Dinic J, Biverståhl H, Mäler L, Parmryd I (2011) Laurdan and di-4-ANEPPDHQ do not respond to membrane-inserted peptides and are good probes for lipid packing. Biochim Biophys Acta 1808: 298–306. 10.1016/j.bbamem.2010.10.002 20937246

[bib13] Flach TL, Ng G, Hari A, Desrosiers MD, Zhang P, Ward SM, Seamone ME, Vilaysane A, Mucsi AD, Fong Y, (2011) Alum interaction with dendritic cell membrane lipids is essential for its adjuvanticity. Nat Med 17: 479–487. 10.1038/nm.2306 21399646

[bib14] Gagné V, Marois L, Levesque JM, Galarneau H, Lahoud MH, Caminschi I, Naccache PH, Tessier P, Fernandes MJ (2013) Modulation of monosodium urate crystal-induced responses in neutrophils by the myeloid inhibitory C-type lectin-like receptor: Potential therapeutic implications. Arthritis Res Ther 15: R73. 10.1186/ar4250 23837669 PMC3978892

[bib15] Giocondi MC, Milhiet PE, Dosset P, Le Grimellec C (2004) Use of cyclodextrin for AFM monitoring of model raft formation. Biophys J 86: 861–869. 10.1016/s0006-3495(04)74161-2 14747321 PMC1303933

[bib16] Hancock JF (2006) Lipid rafts: Contentious only from simplistic standpoints. Nat Rev Mol Cell Biol 7: 456–462. 10.1038/nrm1925 16625153 PMC2782566

[bib17] Hornung V, Bauernfeind F, Halle A, Samstad EO, Kono H, Rock KL, Fitzgerald KA, Latz E (2008) Silica crystals and aluminum salts activate the NALP3 inflammasome through phagosomal destabilization. Nat Immunol 9: 847–856. 10.1038/ni.1631 18604214 PMC2834784

[bib18] Humphrey W, Dalke A, Schulten K (1996) VMD: Visual molecular dynamics. J Mol Graphics 14: 33–38. 10.1016/0263-7855(96)00018-5 8744570

[bib19] Janes PW, Ley SC, Magee AI, Kabouridis PS (2000) The role of lipid rafts in T cell antigen receptor (TCR) signalling. Semin Immunol 12: 23–34. 10.1006/smim.2000.0204 10723795

[bib20] Jin L, Millard AC, Wuskell JP, Dong X, Wu D, Clark HA, Loew LM (2006) Characterization and application of a new optical probe for membrane lipid domains. Biophysical J 90: 2563–2575. 10.1529/biophysj.105.072884 PMC1403187 16415047

[bib21] Kaiser H-J, Lingwood D, Levental I, Sampaio JL, Kalvodova L, Rajendran L, Simons K (2009) Order of lipid phases in model and plasma membranes. Proc Natl Acad Sci U S A 106: 16645–16650. 10.1073/pnas.0908987106 19805351 PMC2757813

[bib22] Lahoud MH, Proietto AI, Ahmet F, Kitsoulis S, Eidsmo L, Wu L, Sathe P, Pietersz S, Chang HW, Walker ID, (2009) The C-type lectin Clec12A present on mouse and human dendritic cells can serve as a target for antigen delivery and enhancement of antibody responses. J Immunol 182: 7587–7594. 10.4049/jimmunol.0900464 19494282

[bib23] Levental I, Levental KR, Heberle FA (2020) Lipid rafts: Controversies resolved, mysteries remain. Trends Cell Biol 30: 341–353. 10.1016/j.tcb.2020.01.009 32302547 PMC7798360

[bib24] Lin X, Shao T, Wen X, Wang M, Wen C, He Z (2020) Combined effects of MSU crystals injection and high fat-diet feeding on the establishment of a gout model in C57BL/6 mice. Adv Rheumatol 60: 52. 10.1186/s42358-020-00155-3 33148336

[bib25] Lorent JH, Levental I (2015) Structural determinants of protein partitioning into ordered membrane domains and lipid rafts. Chem Phys Lipids 192: 23–32. 10.1016/j.chemphyslip.2015.07.022 26241883

[bib26] Lorent JH, Diaz-Rohrer B, Lin X, Spring K, Gorfe AA, Levental KR, Levental I (2017) Structural determinants and functional consequences of protein affinity for membrane rafts. Nat Commun 8: 1219. 10.1038/s41467-017-01328-3 29089556 PMC5663905

[bib27] Luo S-F, Chin C-Y, Ho L-J, Tseng W-Y, Kuo C-F, Lai J-H (2020) Monosodium urate crystals induced ICAM-1 expression and cell–cell adhesion in renal mesangial cells: Implications for the pathogenesis of gouty nephropathy. J Microbiol Immunol Infect 53: 23–32. 10.1016/j.jmii.2017.12.004 29657028

[bib28] Martinon F, Pétrilli V, Mayor A, Tardivel A, Tschopp J (2006) Gout-associated uric acid crystals activate the NALP3 inflammasome. Nature 440: 237–241. 10.1038/nature04516 16407889

[bib29] McIntosh TJ, Vidal A, Simon SA (2003) Sorting of lipids and transmembrane peptides between detergent-soluble bilayers and detergent-resistant rafts. Biophys J 85: 1656–1666. 10.1016/s0006-3495(03)74595-0 12944280 PMC1303339

[bib30] Monticelli L, Kandasamy SK, Periole X, Larson RG, Tieleman DP, Marrink S-J (2008) The MARTINI coarse-grained force field: Extension to proteins. J Chem Theory Comput 4: 819–834. 10.1021/ct700324x 26621095

[bib31] Mu L, Tu Z, Miao L, Ruan H, Kang N, Hei Y, Chen J, Wei W, Gong F, Wang B, (2018) A phosphatidylinositol 4,5-bisphosphate redistribution-based sensing mechanism initiates a phagocytosis programing. Nat Commun 9: 4259. 10.1038/s41467-018-06744-7 30323235 PMC6189171

[bib32] Neumann K, Castiñeiras-Vilariño M, Höckendorf U, Hannesschläger N, Lemeer S, Kupka D, Meyermann S, Lech M, Anders HJ, Kuster B, (2014) Clec12a is an inhibitory receptor for uric acid crystals that regulates inflammation in response to cell death. Immunity 40: 389–399. 10.1016/j.immuni.2013.12.015 24631154

[bib33] Ng G, Sharma K, Ward SM, Desrosiers MD, Stephens LA, Schoel WM, Li T, Lowell CA, Ling C-C, Amrein MW, (2008) Receptor-independent, direct membrane binding leads to cell-surface lipid sorting and Syk kinase activation in dendritic cells. Immunity 29: 807–818. 10.1016/j.immuni.2008.09.013 18993083 PMC2642965

[bib34] Paré G, Vitry J, Merchant ML, Vaillancourt M, Murru A, Shen Y, Elowe S, Lahoud MH, Naccache PH, McLeish KR, (2021) The inhibitory receptor CLEC12A regulates PI3K-Akt signaling to inhibit neutrophil activation and cytokine release. Front Immunol 12: 650808. 10.3389/fimmu.2021.650808 34234773 PMC8256872

[bib35] Pessler F, Mayer CT, Jung SM, Behrens EM, Dai L, Menetski JP, Schumacher HR (2008) Identification of novel monosodium urate crystal regulated mRNAs by transcript profiling of dissected murine air pouch membranes. Arthritis Res Ther 10: R64. 10.1186/ar2435 18522745 PMC2483455

[bib36] Quinn PJ, Wolf C (2009) The liquid-ordered phase in membranes. Biochim Biophys Acta 1788: 33–46. 10.1016/j.bbamem.2008.08.005 18775411

[bib37] Rakhshani H, Dehghanian E, Rahati A (2019) Enhanced GROMACS: Toward a better numerical simulation framework. J Mol Model 25: 355. 10.1007/s00894-019-4232-z 31768713

[bib38] Rock KL, Kataoka H, Lai J-J (2013) Uric acid as a danger signal in gout and its comorbidities. Nat Rev Rheumatol 9: 13–23. 10.1038/nrrheum.2012.143 22945591 PMC3648987

[bib39] Roh SE, Hong YH, Jang DC, Kim J, Kim SJ (2014) Lipid rafts serve as signaling platforms for mGlu1 receptor-mediated calcium signaling in association with caveolin. Mol Brain 7: 9. 10.1186/1756-6606-7-9 24512690 PMC3937055

[bib40] Ruysschaert J-M, Lonez C (2015) Role of lipid microdomains in TLR-mediated signalling. Biochim Biophys Acta 1848: 1860–1867. 10.1016/j.bbamem.2015.03.014 25797518

[bib41] Sezgin E, Levental I, Mayor S, Eggeling C (2017) The mystery of membrane organization: Composition, regulation and roles of lipid rafts. Nat Rev Mol Cell Biol 18: 361–374. 10.1038/nrm.2017.16 28356571 PMC5500228

[bib42] Shi Y (2012) To forge a solid immune recognition. Protein Cell 3: 564–570. 10.1007/s13238-012-2933-5 22717983 PMC4875360

[bib43] Shi Y, Evans JE, Rock KL (2003) Molecular identification of a danger signal that alerts the immune system to dying cells. Nature 425: 516–521. 10.1038/nature01991 14520412

[bib44] Shin S-H, Jeong J, Kim JH, Sohn K-Y, Yoon SY, Kim JW (2020) 1-Palmitoyl-2-Linoleoyl-3-Acetyl-rac-Glycerol (PLAG) mitigates monosodium urate (MSU)-induced acute gouty inflammation in BALB/c mice. Front Immunol 11: 710. 10.3389/fimmu.2020.00710 32395118 PMC7196669

[bib45] Simons K, Ikonen E (1997) Functional rafts in cell membranes. Nature 387: 569–572. 10.1038/42408 9177342

[bib46] Simons K, Sampaio JL (2011) Membrane organization and lipid rafts. Cold Spring Harb Perspect Biol 3: a004697. 10.1101/cshperspect.a004697 21628426 PMC3179338

[bib47] Son YI, Egawa S, Tatsumi T, Redlinger RE, Jr, Kalinski P, Kanto T (2002) A novel bulk-culture method for generating mature dendritic cells from mouse bone marrow cells. J Immunol Methods 262: 145–157. 10.1016/s0022-1759(02)00013-3 11983228

[bib48] Song D, Meng J, Cheng J, Fan Z, Chen P, Ruan H, Tu Z, Kang N, Li N, Xu Y, (2019) Pseudomonas aeruginosa quorum-sensing metabolite induces host immune cell death through cell surface lipid domain dissolution. Nat Microbiol 4: 97–111. 10.1038/s41564-018-0290-8 30510173

[bib49] Štefanová I, Hořejši V, Ansotegui IJ, Knapp W, Stockinger H (1991) GPI-anchored cell-surface molecules complexed to protein tyrosine kinases. Science 254: 1016–1019. 10.1126/science.1719635 1719635

[bib50] van der Goot FG, Harder T (2001) Raft membrane domains: From a liquid-ordered membrane phase to a site of pathogen attack. Semin Immunol 13: 89–97. 10.1006/smim.2000.0300 11308292

[bib51] Varshney P, Yadav V, Saini N (2016) Lipid rafts in immune signalling: Current progress and future perspective. Immunology 149: 13–24. 10.1111/imm.12617 27153983 PMC4981613

[bib52] Wu EL, Cheng X, Jo S, Rui H, Song KC, Dávila-Contreras EM, Qi Y, Lee J, Monje-Galvan V, Venable RM, (2014) CHARMM-GUI Membrane Builder toward realistic biological membrane simulations. J Comput Chem 35: 1997–2004. 10.1002/jcc.23702 25130509 PMC4165794

[bib53] Zidovetzki R, Levitan I (2007) Use of cyclodextrins to manipulate plasma membrane cholesterol content: Evidence, misconceptions and control strategies. Biochim Biophys Acta 1768: 1311–1324. 10.1016/j.bbamem.2007.03.026 17493580 PMC1948080

